# Optimizing belowground interactions in young rubber-banana intercropping via partial organic fertilizer substitution: root competition, soil nutrients, and microbial communities

**DOI:** 10.3389/fpls.2026.1896674

**Published:** 2026-07-31

**Authors:** Dongqi Jin, Hongzhu Yang, Yujie Tan, Lingxuan Qiu, Chunhua Ji, Peisong Zhang, Wei Luo, Zhengzao Cha, Qinghuo Lin, Hailin Liu

**Affiliations:** 1 Haikou Key Laboratory of Soil Health and Nutrient Utilization, Rubber Research Institute, Chinese Academy of Tropical Agricultural Sciences, Haikou, China; 2 Hainan Institute of National Park/Key Laboratory for National Park Protection and Development of Hainan Province, Haikou, China; 3 Danzhou Soil Environment of Rubber Plantation, Hainan Observation and Research Station, Danzhou, China; 4 School of Tropical Agriculture and Forestry, Hainan University, Haikou, China; 5 Sanya Research Institute, Chinese Academy of Tropical Agricultural Sciences, Sanya, China

**Keywords:** belowground competition, intercropping, microbial community, organic fertilizer, root distribution, rubber-banana system

## Abstract

**Aims:**

Optimal fertilization strategies for rubber tree-banana intercropping systems and their comprehensive impacts on belowground interactions remain inadequately characterized. This study systematically evaluated the effects of different fertilization regimes on root distribution, interspecific competition, and rhizosphere properties.

**Methods:**

The experimental design included four treatments: (1) rubber monoculture with conventional fertilization (CK); and three rubber-banana intercropping systems with different fertilization regimes: (2) conventional chemical fertilization (M1), (3) 30% organic nitrogen substitution (M2), and (4) 45% controlled-release nitrogen combined with chemical nitrogen (M3). Thus, the effect of intercropping per se can be assessed by comparing CK with M1, while the effect of fertilization strategies within intercropping is evaluated by comparing M1, M2, and M3.

**Results:**

Results demonstrated that the M2 treatment produced the most significant improvements, enhancing rubber tree height by 16.26% and stem girth by 8.17% compared to CK, while simultaneously supporting optimal banana growth. During the banana bud development stage, M2 increased rubber root biomass in the 0–20 cm and 20–40 cm layers by 66.91%–115.84% and 140.08%–625.47%, respectively, compared to other treatments, and promoted banana root length density mainly in the surface layer (0–20 cm) by 79.98%–88.50%. Soil available N under M2 was elevated by 14.43%–26.49% across different soil layers at harvest, and available P increased by 12.72% in the 0–20 cm layer, while soil organic matter increased by 7.77%. Microbial community shifts showed strong positive correlations with increased soil nutrients; for example, Firmicutes was enriched by 131.00%, and key taxa such as Proteobacteria and Bacteroidota were also significantly increased and positively correlated with root development. Importantly, M2 reduced root competition intensity (UICII) by 22.69%–31.65% during vigorous growth and bud development stages.

**Conclusions:**

Our integrated analysis demonstrates that substituting 30% of chemical nitrogen with organic nitrogen appears promising under the experimental conditions of this study, as it simultaneously enhances plant productivity, alleviates belowground competition, and improves soil ecological conditions. Therefore, this study provides a scientifically-grounded and potentially sustainable management strategy for enhancing the productivity and ecological sustainability of rubber tree-banana intercropping systems.

## Highlights

- M2 (30% organic nitrogen substitution) optimally promotes the growth of both rubber trees and bananas.
- M2 optimizes root spatial-temporal distribution and alleviates belowground competition.
- M2 enhances soil nitrogen, phosphorus, and organic matter content.
- M2 increases rhizosphere microbial diversity and enriches beneficial taxa.
- M2 is recommended as a superior fertilization strategy for the intercropping system.

## 1 Introduction

Natural rubber, a key polymer material with exceptional properties, is widely utilized across various industrial applications. It is primarily sourced from rubber tree (*Hevea brasiliensis*) plantations (Oouchi et al., 2019; Silva et al., 2023). A significant challenge in global rubber production lies in the fact that rubber trees require an initial growth period of approximately six years before they begin to produce latex, during which no income is generated (Michels et al., 2012; Rodrigo et al., 2004). To address this income gap in the early stages of rubber production, farmers have adopted intercropping practices, integrating rubber trees with short−term annual and perennial crops such as tea, coffee, and bananas (Rodrigo et al., 2004; Wu et al., 2016a; Yuan et al., 2024). Research suggests that native rubber tree agroforestry systems intercropped with other crops not only diversify income streams but also enable multi−variety and multi−level combinations, thereby optimizing resource utilization, including sunlight, soil moisture, and nutrients (Qi et al., 2024, 2021).

Intercropping offers a potentially effective strategy for enhancing farm profitability by increasing crop yields, improving soil fertility, and reducing environmental impact (Li and Lin, 2021; Yang et al., 2020). The full realization of these advantages relies critically on rational nutrient management and its regulation of root systems. The application of appropriate fertilization strategies and effective nutrient management can stimulate root growth and influence root morphology and architecture (Huang et al., 2022; Liu et al., 2024; Mengmeng et al., 2021). Moreover, the dynamic interplay between fertilization treatments and rhizosphere soil microorganisms also drive key biogeochemical processes, including nitrogen (N) fixation, phosphorus (P) solubilization, and carbon (C) cycling (Asghar et al., 2024; Roohi et al., 2020; Yu et al., 2020). For instance, legume−based intercropping systems demonstrate significant shifts in microbial diversity and abundance under varying N and P fertilization regimes, with low−N treatments often favoring nitrogen−fixing bacteria like *Bradyrhizobium* and *Allorhizobium−Neorhizobium−Pararhizobium−Rhizobium* (Cheng et al., 2023). Conversely, excessive N application can suppress these beneficial taxa, highlighting the need for balanced fertilization strategies to maintain microbial−mediated ecosystem services (A et al., 2021; Cheng et al., 2023). However, if the interaction between tree and crop root systems becomes excessively competitive, resource shortages may arise, undermining the potential benefits of intercropping and preventing optimal nutrient utilization. The competition for soil resources is influenced by the spatial distribution of roots, and the intensity of this competition can alter root spatial arrangement (Cardinael et al., 2015). Therefore, optimizing root system architecture may help regulate interspecific interactions within cropping systems (Liu et al., 2023). In intercropping systems, strategic nitrogen application can modify crop growth dynamics by enhancing or mitigating interspecific competition (Gong et al., 2021).

Research demonstrates that the nutrient release characteristics of organic nitrogen and controlled−release nitrogen, when applied in appropriate proportions alongside chemical nitrogen, can improve both crop yield and stability. Innovative fertilization approaches, such as integrated organic−inorganic amendments (e.g., NPK−enriched compost or pig manure), have been shown to enhance microbial biomass and enzyme activities (e.g., sucrase, β−1,4−glucosidase, and phosphatases), which are critical for nutrient mobilization and soil health (Jiang et al., 2022; Roohi et al., 2020; Wu et al., 2016b). It is critical for fertilization systems to adapt nitrogen management practices to optimize the temporal and spatial relationship between nitrogen supply and crop demand (Hu et al., 2020). In intercropping systems involving rubber trees and calla lilies, calla lilies enhance their competitive advantage for soil nitrogen by increasing root length and preferentially allocating root biomass (Li et al., 2018). Although rubber trees possess deep, extensive root systems, intercropped species typically concentrate their roots closer to the soil surface, thereby increasing competition for nutrients and water (Yang et al., 2021). Root competition between rubber trees and intercropped species presents a significant challenge in assessing the viability of rubber agroforestry systems (Wu et al., 2016a). The functional redundancy and niche differentiation of microbial communities under intercropping further complicate their response to fertilization. For example, ammonia−oxidizing archaea (AOA) and bacteria (AOB) exhibit distinct preferences for soil microhabitats, with their abundances strongly correlated with soil pH and organic C content (Han et al., 2020; Zheng et al., 2022b). Prior to the widespread implementation of rubber tree agroforestry, selecting intercropping species with complementary root strategies to mitigate competition is essential (Langenberger et al., 2016). Thus, effective nitrogen fertilizer management strategies in rubber−based agroforestry systems can significantly enhance soil nutrient levels, optimize the spatial and temporal distribution of roots, and promote the sustainable development of the system.

Banana (*Musa acuminata*), a staple food crop grown in over 130 countries, is of particular importance in China as a tropical fruit. While some studies have examined the intercropping of rubber trees and bananas, research on the spatial and temporal distribution of root morphology, soil nutrient dynamics, and root competition under various fertilization patterns remains limited. Furthermore, how the rhizosphere microbial community in this system responds to partial organic nitrogen substitution, and how microbial processes interact with root distribution and soil nutrients, is currently unclear. This study hypothesized that partial substitution of chemical N with organic N would (1) optimize the spatiotemporal distribution of roots in the rubber−banana intercropping system, thereby reducing interspecific root competition; (2) improve soil nutrient availability, particularly for N and P; and (3) enhance the diversity and structure of rhizosphere microbial communities, which in turn mediate positive effects on plant growth and soil fertility. To test these hypotheses, we compared a rubber monoculture (CK) with three intercropping fertilization regimes: conventional fertilization (M1), 30% organic N substitution (M2), and 45% controlled−release N (M3). The specific objectives were: (a) to quantify the spatiotemporal distribution of root length density, root surface area density, and root biomass; (b) to calculate the underground interspecific competition intensity index (UICII); (c) to measure soil available N, P, K, and organic matter; and (d) to characterize the bacterial and fungal communities in rhizosphere soil using high−throughput sequencing. This study aims to provide practical recommendations for optimizing N fertilizer management in young rubber−banana intercropping systems, thereby establishing a pathway to sustainable development.

## 2 Materials and methods

### 2.1 Experimental site

**Figure 1:**
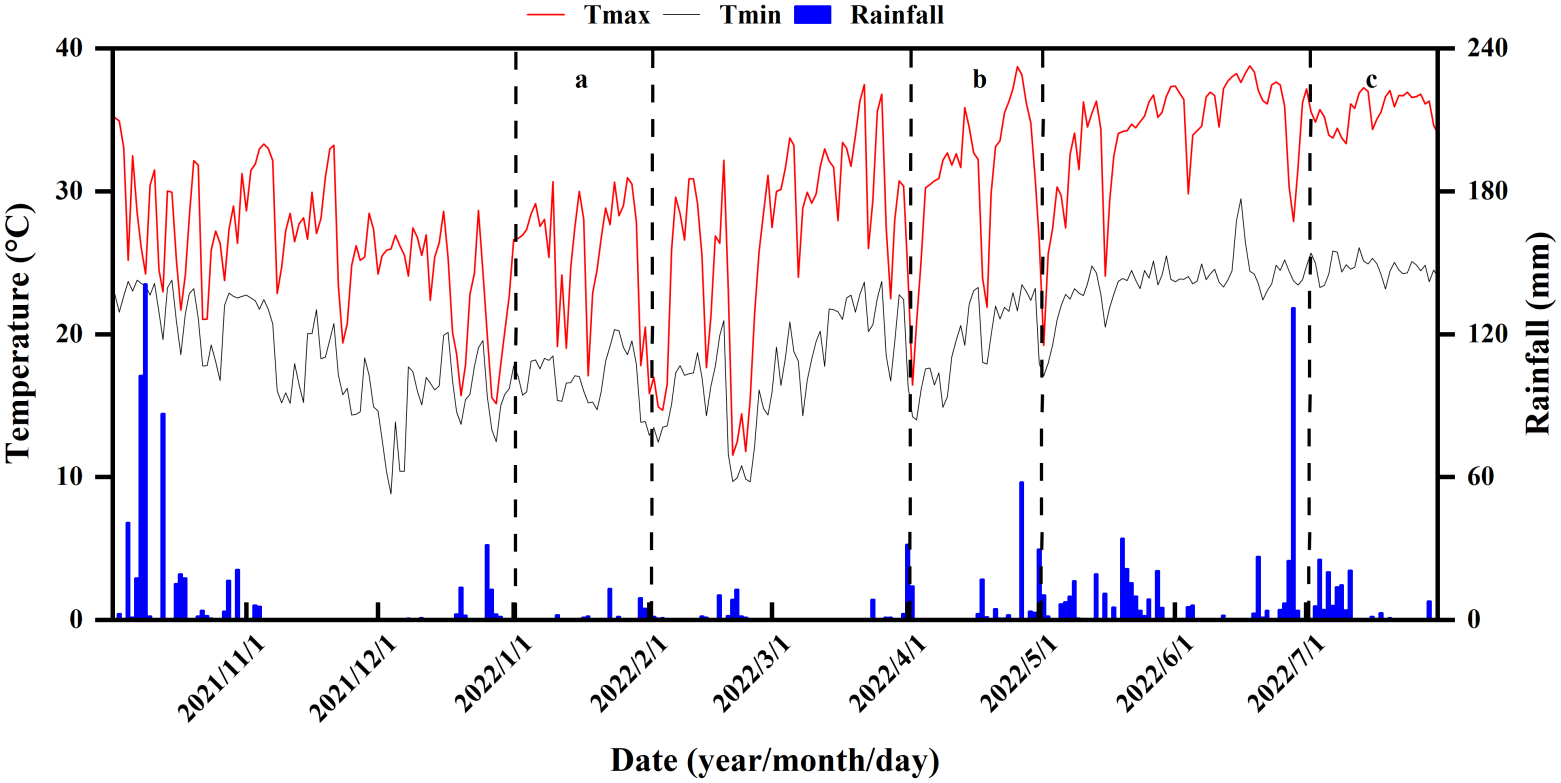
Daily temperature and rainfall during the banana growth stage. **(a)** represents the vigorous stage of banana, **(b)** represents the bud pregnancy stage of banana, **(c)** represents the harvest stage of banana.

Field experiments were conducted from October 2021 to August 2022 at the Tropical Fruit Tree Variety Improvement Base, Tropical Crops Genetic Resources Institute, Chinese Academy of Tropical Agricultural Sciences, located in Danzhou County, Hainan Province, China (109.5°E, 19.5°N). The experimental site is situated in a hot and humid climate, with a mean annual temperature of 23.3 °C and annual precipitation of 1826 mm (**Figure 1**).

### 2.2 Experimental materials

The soil at the experimental site was classified as brick−red loam, with a pH of 5.16, organic matter content of 8.74 g·kg^−1^, total nitrogen content of 0.76 g·kg^−1^, and available nitrogen content of 100.85 mg·kg^−1^. The available phosphorus content was 7.32 mg·kg^−1^, and the available potassium content was 90.83 mg·kg^−1^ at the beginning of the experiment. The fertilizers used in the experiment included diammonium phosphate (N ≥ 20.8%, P_2_O_5_ ≥ 53%), urea (N ≥ 46%), potassium dihydrogen phosphate (P_2_O_5_ ≥ 52%, K_2_O ≥ 34%), potassium chloride (K_2_O ≥ 60%), polyurethane−coated urea with a fertilizer efficacy period of three months (N ≥ 43%), organic fertilizer (organic matter ≥ 45%, N−P_2_O_5_−K_2_O ≥ 5%), and water−soluble organic fertilizer (organic matter ≥ 320 g/L; N− P_2_O_5_− K_2_O ≥ 80 g/L). The seedlings tested included one−year−old tissue culture rubber tree Reyan 7−33−97 seedlings and Jiali banana tissue culture seedlings with 8–9 leaves.

### 2.3 Experimental design

The experiment employed a single−factor random block design with four treatments: monoculture rubber trees (CK), rubber tree−banana intercropping with conventional fertilization (M1), rubber tree−banana intercropping with a 30% organic nitrogen substitution treatment (M2), and rubber tree−banana intercropping with a 45% controlled−release nitrogen fertilizer combined with conventional chemical nitrogen treatment (M3). Each plot contained 7 rubber trees planted at a spacing of 2 m × 4 m × 20 m, and 54 banana plants spaced 1.5 m × 1.5 m. Each treatment was replicated three times, yielding a total of 12 experimental plots, each with an area of 126 m^2^ (10.5 m × 12.0 m). In the initial year, the rubber trees in all treatments received the following fertilizer application: N 50 g·tree^−1^, P_2_O_5–_20 g·tree^−1^, K_2_O 55 g·tree^−1^. The monoculture rubber trees (CK) received the same conventional fertilization as the intercropped rubber trees in the M1 treatment. For the banana plants, treatments M1, M2, and M3 received a total nutrient dosage throughout their growth period of N 180 g, P_2_O_5–_80 g, and K_2_O 400 g. Nitrogen (N) was applied according to the following distribution: 10% at the seedling stage, 20% during vigorous growth, 20% at flower bud differentiation, 25% during bud development, 20% at bud emergence, and 5% at fruit expansion. Phosphorus (P) application followed this schedule: 20% at the seedling stage, 20% during vigorous growth, 20% at flower bud differentiation, 20% during bud development, 15% at bud emergence, and 5% at fruit expansion. Potassium (K) was distributed as follows: 5% at the seedling stage, 10% during vigorous growth, 20% at flower bud differentiation, 25% during bud development, 30% at bud emergence, and 10% at fruit expansion. In the M2 treatment, 30% of the total N for bananas was derived from organic fertilizer (with 50% as powdered organic fertilizer at the base and 50% as water−soluble organic fertilizer), and the remaining 70% was supplied as urea (chemical N), in line with the N application schedule for each growth stage. In the M3 treatment, 45% of the total N was supplied as polyurethane−coated urea, and the remaining 55% was supplied as conventional urea. The M1 and M2 treatment employed fertigation, while the M3 treatment involved six split applications of fertilizers via furrow application. Detailed quantities of fertilizer for banana cultivation are presented in **Supplementary Table 1**.

### 2.4 Tree height and stem girth

The height of each rubber tree and banana plant was measured at the banana harvest stage using a hypsometer. Stem girth was recorded 100 cm above the soil surface for each plant within the plot (N = 5).

### 2.5 Leaf chlorophyll content

During the banana harvest stage, mature leaves from the second leaf of both rubber trees and banana plants were selected for sampling. A 1:1 mixture of acetone and ethanol was used for a 24−hour light−proof extraction. The photosynthetic pigment content, including chlorophyll a, chlorophyll b, and total chlorophyll, was quantified using a UV−visible spectrophotometer (Wellburn, 1994).

### 2.6 Roots and soil sampling and analysis

**Figure 2:**
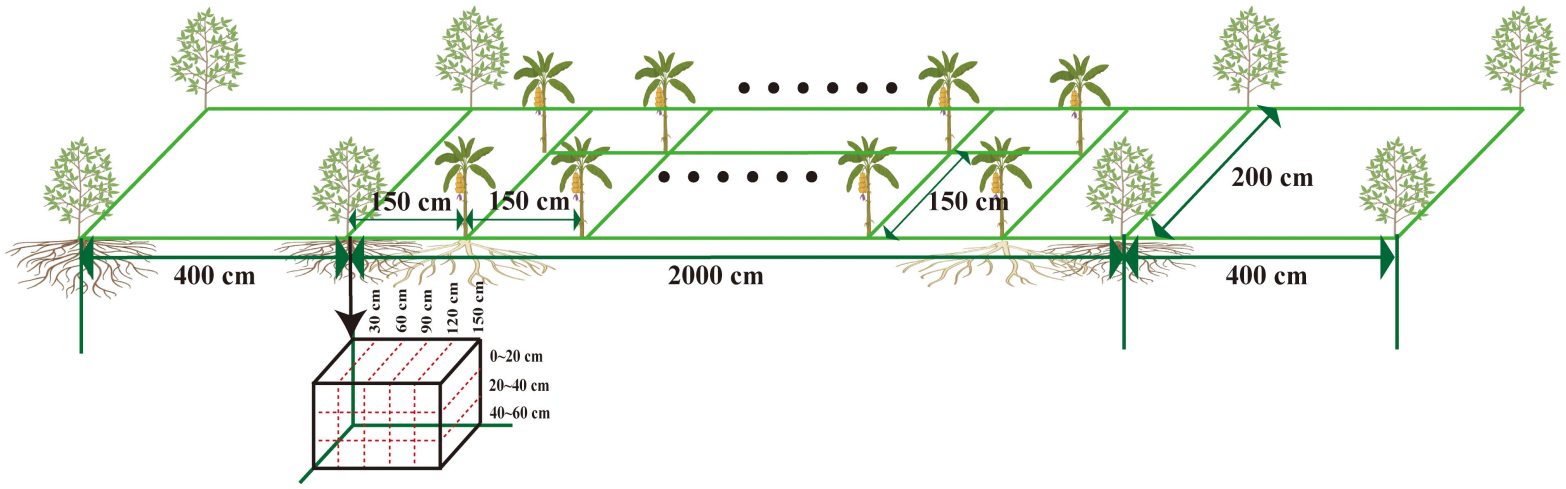
Sketch of sampling procedure for rubber tree and banana soil under each treatment.

The rubber trees selected from each plot showed consistent growth, and a stratified excavation method was employed to sample the intercropping zones of rubber trees and bananas in the vigorous growth stage of bananas, the bud development stage, and the harvest stage. These stages were defined as follows: (1) vigorous growth stage is characterized rapid elongation of leaves and pseudostem; (2) bud development stage – from flower bud differentiation to inflorescence emergence (bud emergence); (3) harvest stage – from fruit filling to fruit maturity. The rubber tree−banana intercropping area was divided into five equal sections, each 30 cm apart, with reference to the rubber tree. Sampling blocks, each measuring 60 cm × 30 cm × 20 cm (volume = 3.6 × 10^4^ cm^3^), were excavated at depths of 0−20 cm, 20−40 cm, and 40−60 cm (**Figure 2**). Root systems were carefully retrieved from each soil block. The bulk soil loosely adhering to the roots was removed and mixed to form a composite non−rhizosphere soil sample. This soil was then sealed in bags and transported to the laboratory for the determination of available nitrogen, available phosphorus, available potassium, and organic matter content. All collected roots were thoroughly rinsed with water and stored at –4 °C for further analysis. After carefully retrieving the root systems, the soil that remained tightly attached to the roots was defined as rhizosphere soil. This soil was gently brushed off from the roots using a sterile brush, passed through a 2–mm sieve, and then immediately frozen in liquid nitrogen, transferred to a −80 °C ultra−low temperature freezer upon arrival at the laboratory, and subsequently used for microbial analysis.

### 2.7 Determination of morphological measurements and soil nutrients

#### 2.7.1 Root morphological measurements

The root shape index was determined by gently drying the clean rubber and banana roots using soft paper, followed by measuring their fresh weight. The roots were then scanned using an Epson scanner, and the obtained data, including root length and root surface area, were analyzed with WinRHIZO™ software (Regent Instruments Inc., Quebec, Canada).

#### 2.7.2 Data processing

The root length density (RLD, cm·cm^−3^) was calculated using (**Equation 1**).

(1) RLD=RL/SV

In this equation, RL represents root length (cm) and SV denotes soil volume (cm^3^). The root surface area density (RSAD, cm^2^·cm^−3^) was determined using (**Equation 2**).

(2) RSAD=RSA/SV

In this equation, RSA refers to the root surface area (cm^2^) and SV is the soil volume (cm^3^).

The intensity of soil nutrient competition between the roots of rubber trees and bananas was quantified by quantifying the degree of overlap between the ecological niches occupied by each species (Levins, 1968). The underground interspecific competition intensity index (UICII) was calculated by (**Equation 3**).

(3) $$UICII=\frac{\sum_{i=1}^{n}P_{Ri}P_{Bi}}{\sqrt{\sum_{i=1}^{n}P_{Ri}^{2}\sum_{i=1}^{n}P_{Bi}^{2}}}$$

The formula of *UICII* (0 ≤ UICII ≤ 1) is as follows (Pianka, 1973): where *n* represents the number of soil layers, and *P_Ri* and *P_Bi* denote the root proportion of rubber trees and bananas, respectively, in the *i* th soil layer, relative to the total root distribution across all layers (soil depth 0−60 cm, distance from the tree 0−150 cm). This index quantifies the degree of overlap between the ecological niches occupied by each species; it indicates root distribution overlap, which reflects potential competition for soil resources, but does not directly measure nutrient uptake or depletion.

#### 2.7.3 Soil nutrition measurements

Soil properties were determined as follows: soil organic matter content was quantified using the potassium dichromate volumetric method with external heating; available nitrogen was measured through distillation using a semi−micro Kjeldahl apparatus; available phosphorus was determined by HCl−NH_4_F molybdenum blue colorimetry; and available potassium was analyzed *via* flame photometry following ammonia acetate extraction. These analytical methods were adapted from Bao (2000).

#### 2.7.4 Microbiome analysis

The bacterial and fungal community structures in the rhizosphere soil of rubber trees and bananas were analyzed using 16S rRNA and ITS amplicon sequencing, respectively. The detailed experimental procedure was as follows:

##### 2.7.4.1 DNA extraction and amplicon sequencing

Total DNA was extracted from the collected rhizosphere soil samples using a commercial kit. The V3−V4 hypervariable region of the bacterial 16S rRNA gene was amplified using the specific primers 338F (5′−ACTCCTACGGGAGGCAGCAG−3′) and 806R (5′−GGACTACHVGGGTWTCTAAT−3′). The ITS1 region of the fungal ITS gene was amplified using the primers ITS1F (5′−CTTGGTCATTTAGAGGAAGTAA−3′) and ITS2R (5′−GCTGCGTTCTTCATCGATGC−3′). The PCR reactions were carried out in a 15 μL mixture containing Phusion^®^ High−Fidelity PCR Master Mix, 0.2 μM of each forward and reverse primer, and approximately 10 ng of template DNA. The thermal cycling conditions consisted of an initial denaturation at 98 °C for 1 min; followed by 30 cycles of denaturation at 98 °C for 10 s, annealing at 50 °C for 30 s, and elongation at 72 °C for 30 s; with a final extension at 72 °C for 5 min.

##### 2.7.4.2 Library construction and sequencing

The PCR products were detected by electrophoresis on a 2% agarose gel. Subsequently, the PCR products were pooled in equimolar ratios and purified. Sequencing libraries were constructed, and the quality of the libraries was assessed using Qubit for quantification and a bioanalyzer for size distribution detection. The qualified libraries were finally paired−end sequenced on an Illumina platform according to the effective library concentration and the required data volume.

##### 2.7.4.3 Bioinformatics analysis

Raw reads were quality−filtered and trimmed using Trimmomatic (v0.39). Chimeric sequences were removed with VSEARCH (v2.15.0). High−quality reads were clustered into operational taxonomic units (OTUs) at 97% sequence identity using UPARSE (v11.0). Taxonomic assignment was performed against the SILVA database (v138) for bacteria and the UNITE database (v8.2) for fungi. Alpha diversity (Shannon and Chao1 indices) was calculated using QIIME2 (v2020.2).

### 2.8 Statistical analyses

All data are presented as mean ± standard error (SE) from three biological replicates (n=3). Statistical analysis was performed using one−way ANOVA followed by Duncan’s multiple range test (*p* < 0.05). All computations, including comparisons of the relative abundances of microbial taxa and alpha diversity indices (Shannon and Chao1), were carried out using SPSS 29.0 (IBM Corporation, USA). Experimental figures were generated using Origin 2021 (Origin Lab, USA). For microbial community analysis, Spearman correlation analysis was conducted to evaluate relationships between the relative abundance of dominant microbial phyla and root morphological or soil nutrient parameters. These analyses and associated visualizations were performed using R software (version 4.2.0) with the vegan and ggplot2 packages.

## 3 Results

### 3.1 Plant growth and leaf chlorophyll content

**Figure 3:**
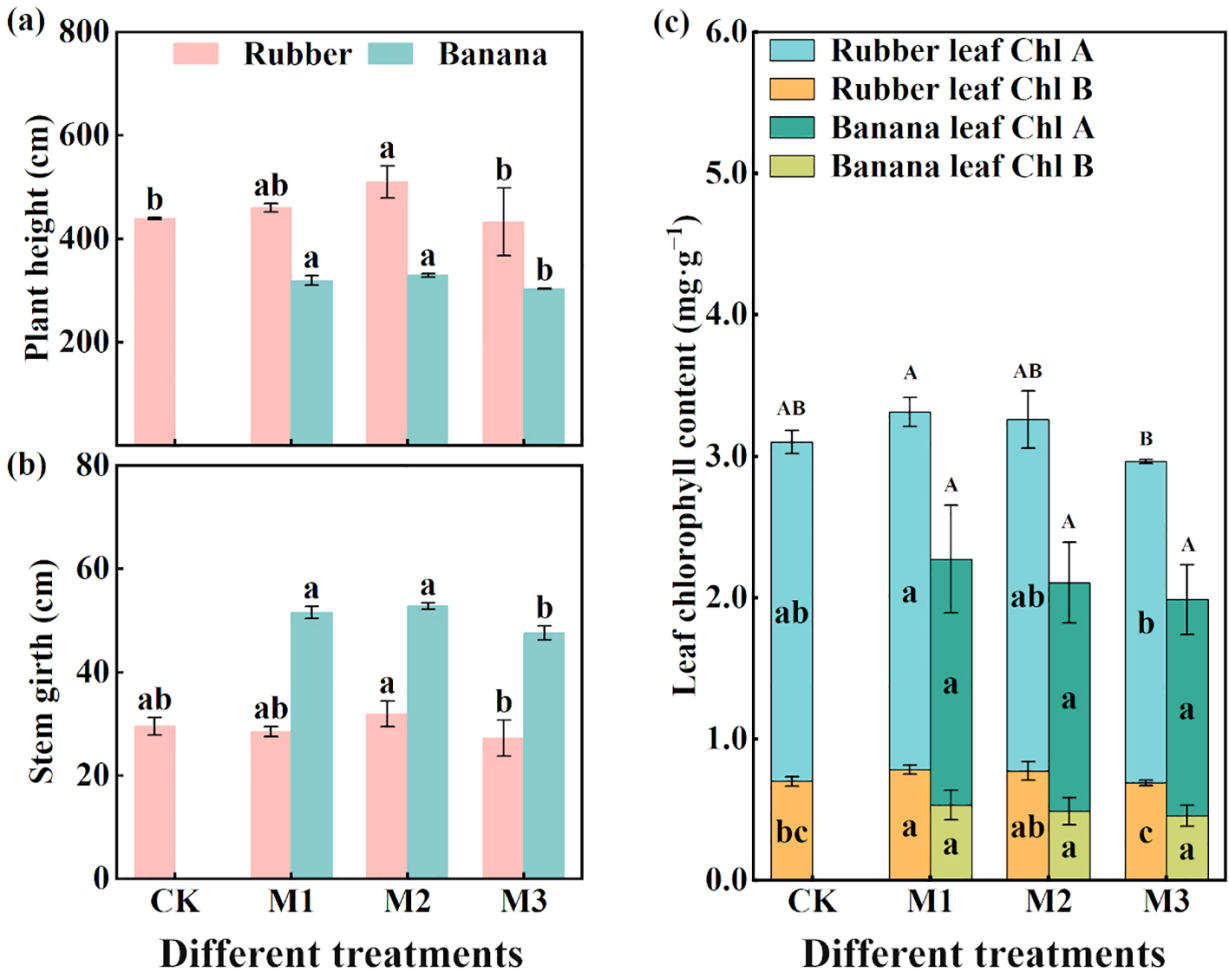
Effects of different fertilization regimes on plant growth and leaf chlorophyll content. **(a)** Plant height, **(b)** stem girth, and **(c)** total chlorophyll content of rubber trees (left panels) and banana plants (right panels). CK: rubber monoculture with conventional fertilization; M1: intercropping with conventional fertilization; M2: intercropping with 30% organic N substitution; M3: intercropping with 45% controlled−release (N) Error bars represent standard errors (SE, n=3). Different lowercase letters (a–c) indicate significant differences among treatments within the same species (p < 0.05, Duncan's test).

The rubber tree/banana intercropping system with different fertilization strategies significantly influenced both plant growth and leaf chlorophyll content. For rubber trees, plant height increased by 4.83% under M1 and 16.26% under M2 compared to the monoculture control (CK), with M2 showing a significant increase (*p* < 0.05), while M3 resulted in a non−significant reduction (**Figure 3A**). Stem girth of rubber trees increased by 8.17% under M2 compared to CK, whereas M1 and M3 showed no significant changes (**Figure 3B**). For bananas, both M1 and M2 significantly enhanced plant height by 5.32% and 8.66%, respectively, compared to M3 (**Figure 3A**), and increased stem girth by 8.34% and 10.93%, respectively (**Figure 3B**). In bananas, both M1 and M2 significantly enhanced plant height by 5.32% and 8.66%, respectively, compared to M3 (**Figure 3A**), while stem girth increased by 8.34% and 10.93% under the same treatments (**Figure 3B**). Regarding leaf chlorophyll content, rubber trees under M1 and M2 showed increased chlorophyll a by 5.42% and 3.61%, and total chlorophyll by 4.94% and 6.77% compared to CK, while M3 decreased total chlorophyll by 4.62% (**Figure 3C**). No significant differences in chlorophyll content were observed in banana leaves across treatments (**Figure 3C**). Although both M1 and M2 treatments promoted plant growth compared to CK, the M2 treatment showed superior or comparable effects on most parameters, particularly in rubber tree height and banana stem girth.

### 3.2 Spatial and temporal distribution of root system

#### 3.2.1 Root biomass

**Figure 4:**
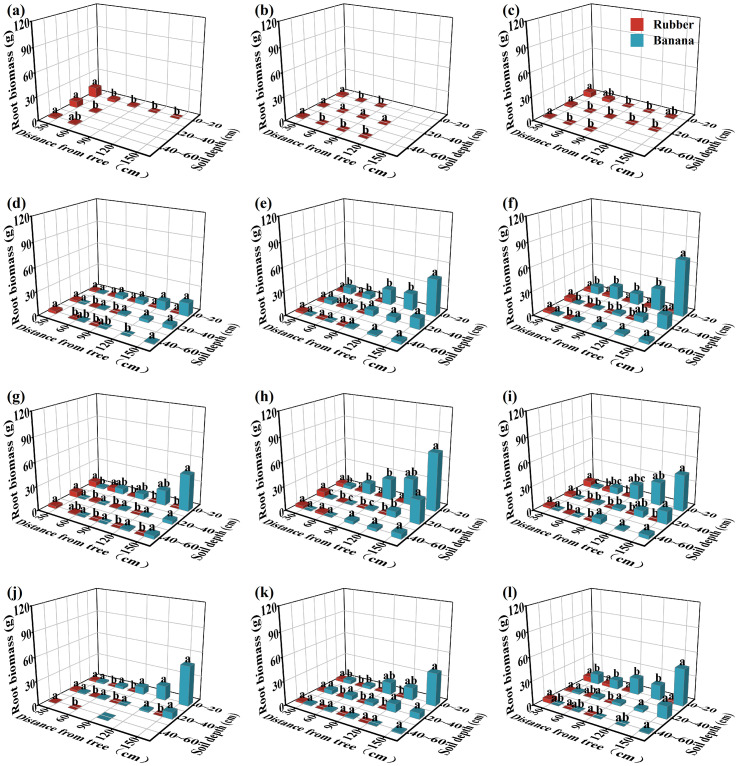
Distribution of root biomass. Vigorous growth stage **(A, D, G, J)**, bud development stage **(B, E, H, K)**, harvest stage **(C, F, I, L)**, from top to bottom are CK, M1, M2, M3. CK, control treatment as young rubber tree monoculture; M1, conventional chemical fertilizer treatment; M2, 30% organic nitrogen substitution treatment; M3, controlled released nitrogen fertilizer combined with 45% treatment.

The temporal and spatial distribution of root biomass for rubber tree monoculture and three different fertilization treatments in rubber−banana intercropping systems is presented in **Figure 4**. Over time, from the banana vigorous growth stage to the bud development stage, the rubber tree root biomass decreased in the CK, M1, and M2 treatments, while it increased in the M3 treatment. At the banana bud development stage, the rubber root biomass was highest in the M2 treatment, reaching 21.78 g. From the banana bud development stage to the banana harvest stage, rubber root biomass generally increased in all treatments, except for a slight decrease in the M2 treatment. Nonetheless, by the banana harvest stage, the rubber tree root biomass in the M2 treatment remained the highest, at 19.70 g. In terms of banana root biomass, the M1 and M3 treatments peaked during the harvest period, reaching 177.69 g and 140.48 g, respectively, while the M2 treatment showed its peak at 203.02 g during the bud development stage. Regarding vertical spatial variation, during the banana bud development stage, rubber tree root biomass in the M2 treatment was higher than in other treatments. Specifically, in the 0−20 cm soil layer, biomass increased by 66.91% to 115.84%, while in the 20−40 cm layer, it increased by 140.08% to 625.47%. In the 40−60 cm layer, the increase ranged from 113.10% to 239.40%. Comparatively, banana root biomass in the M2 treatment in the 0−20 cm layer increased by 47.51% to 85.67% over the M1 and M3 treatments, while in the 20−40 cm layer, the increase was 11.10%, and in the 40−60 cm layer, it increased by 20.17% and 92.80%, respectively. In the horizontal distribution, during the banana harvest stage, within a 0−30 cm radius from the rubber tree, the rubber tree root biomass in the M2 treatment increased by 7.09% to 29.17% compared to other treatments.

#### 3.2.2 Root length density

**Figure 5:**
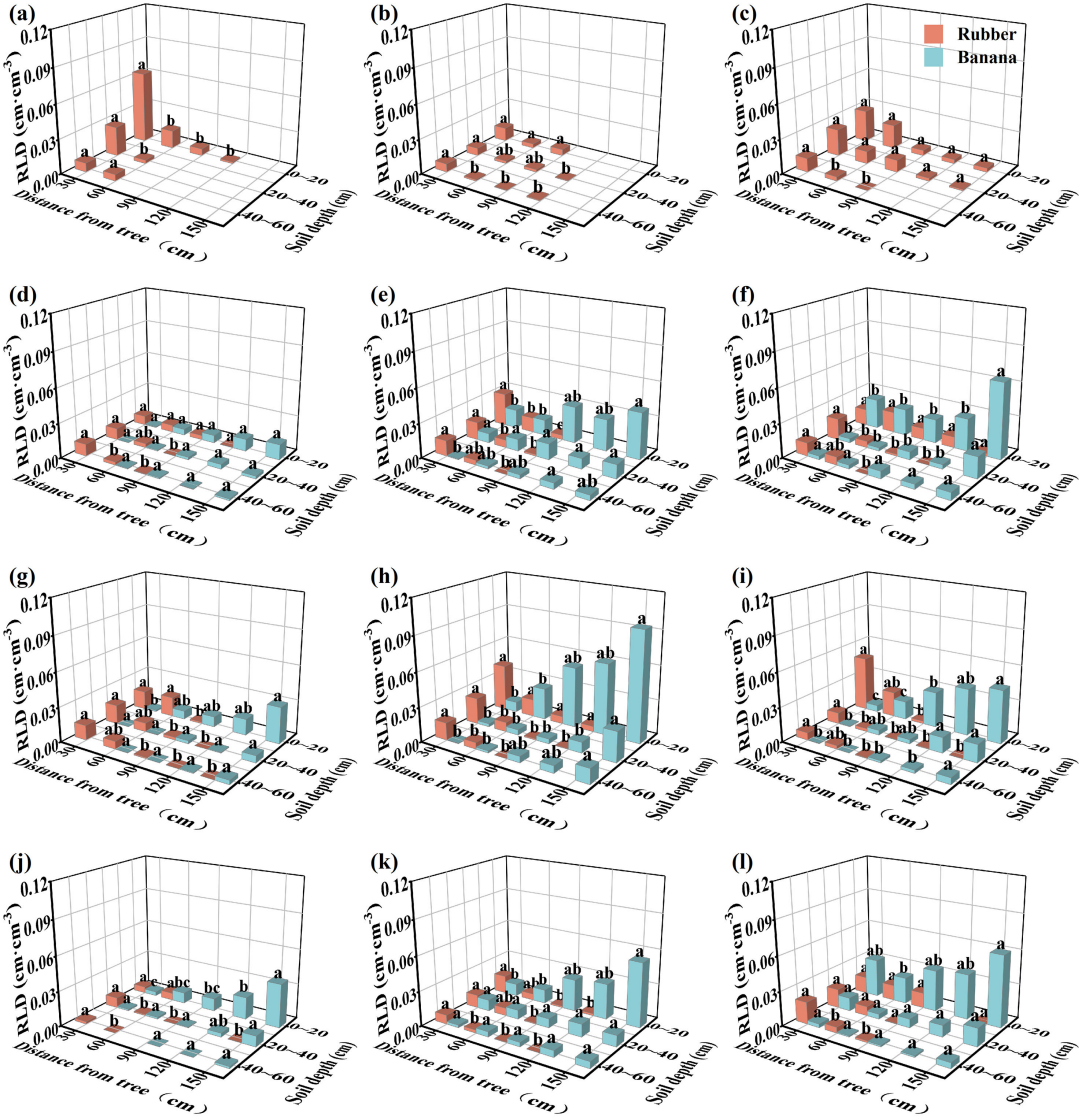
Distribution of root length destiny (RLD). Vigorous growth stage **(A, D, G, J)**, bud development stage **(B, E, H, K)**, harvest stage **(C, F, I, L)**, from top to bottom are CK, M1, M2, M3. CK, control treatment as young rubber tree monoculture; M1, conventional chemical fertilizer treatment; M2, 30% organic nitrogen substitution treatment; M3, controlled released nitrogen fertilizer combined with 45% treatment.

The temporal and spatial distribution of RLD in rubber tree monoculture and three different fertilization modes in rubber−banana intercropping systems is presented in **Figure 5**. In terms of temporal variation, from the vigorous growth stage to the bud development stage of banana, the total RLD of rubber trees in the CK treatment decreased, while in the M1, M2, and M3 treatments, it increased. At the bud development stage, the total RLD of rubber trees in the M2 treatment peaked at 0.364 cm·cm^−3^. Moving from the bud development stage to the harvest stage, the total RLD of rubber trees increased in the CK, M1, and M3 treatments, while it decreased in the M2 treatment. At the harvest stage, the highest total RLD of rubber trees was observed in the CK treatment, at 0.380 cm·cm^−3^. Regarding banana RLD, the total RLD of the M1 and M3 treatments showed an increasing trend throughout banana growth, reaching peaks of 0.673 cm·cm^−3^ and 0.764 cm·cm^−3^, respectively, at the harvest stage. In contrast, the highest total RLD for bananas in the M2 treatment occurred at the bud development stage, at 0.963 cm·cm^−3^. In terms of vertical spatial variation, during the banana bud development stage, in the 0−20 cm soil layer, the total RLD of rubber trees in the M2 treatment increased by 213.44%, 44.78%, and 171.84% compared to the CK, M1, and M3 treatments, respectively. The total banana RLD in the M2 treatment increased by 88.50% and 79.98% compared to the M1 and M3 treatments, respectively. In the horizontal distribution, during the banana bud development stage, within a 0−30 cm distance from the rubber tree, the RLD of rubber trees in the M2 treatment increased by 203.37%, 34.53%, and 124.02% compared to the CK, M1, and M3 treatments, respectively. At a distance of 120−150 cm from the rubber tree, the RLD of bananas in the M2 treatment increased by 135.52% and 89.12% compared to the M1 and M3 treatments.

#### 3.2.3 Root surface area density

**Figure 6:**
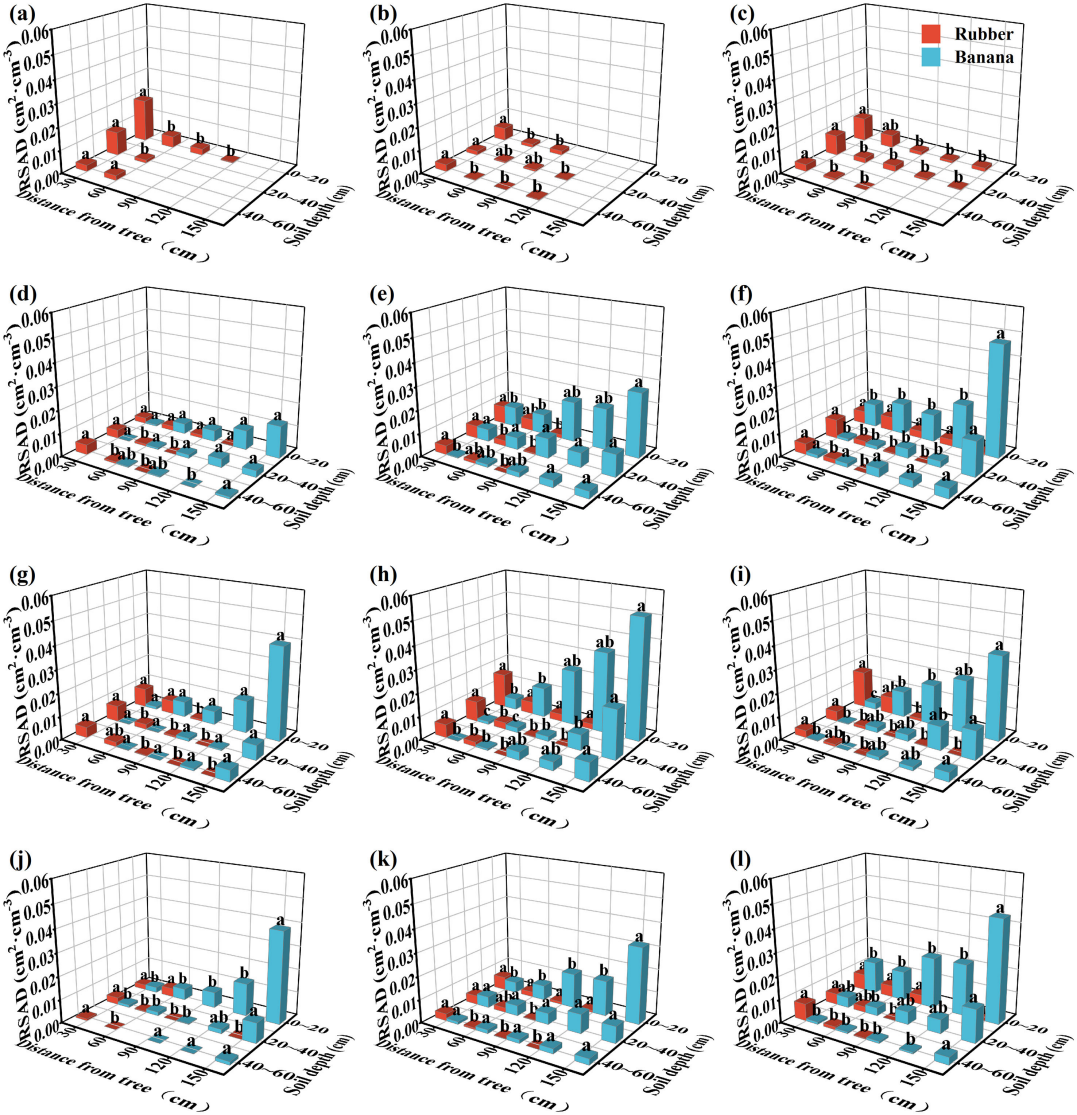
Distribution of root surface area destiny (RSAD). Vigorous growth stage **(A, D, G, J)**, bud development stage **(B, E, H, K)**, harvest stage **(C, F, I, L)**, from top to bottom are CK, M1, M2, M3. CK, control treatment as young rubber tree monoculture; M1, conventional chemical fertilizer treatment; M2, 30% organic nitrogen substitution treatment; M3, controlled released nitrogen fertilizer combined with 45% treatment.

The temporal and spatial distribution of RSAD in rubber tree monoculture and three different fertilization modes in rubber−banana intercropping systems is illustrated in **Figure 6**. From the banana vigorous growth stage to the bud development stage, the RSAD of rubber trees decreased in the CK treatment, while it increased in the M1, M2, and M3 treatments. The highest RSAD value for rubber trees during the bud development stage was recorded in the M2 treatment, at 0.138 cm^2^·cm^−3^. At the harvest stage, the RSAD of rubber trees in the M2 treatment slightly decreased, while it increased in the other treatments. The RSAD in the CK treatment was 0.116 cm^2^·cm^−3^, the highest among all treatments. Regarding banana RSAD, the peak values in the M1 and M3 treatments occurred during the harvest stage, at 0.424 cm^2^·cm^−3^ and 0.469 cm^2^·cm^−3^, respectively, while the maximum RSAD in the M2 treatment was observed during the bud development stage, at 0.534 cm^2^·cm^−3^. From the perspective of vertical spatial variation during the banana bud development stage, in the 0−20 cm soil layer, the RSAD of rubber trees in the M2 treatment increased by 187.48%, 77.08%, and 162.10% compared to the CK, M1, and M3 treatments, respectively. Additionally, the RSAD of banana plants in the M2 treatment was higher than in the M1 and M3 treatments, with increases of 58.48% and 72.63%, respectively. In the horizontal distribution, at a distance of 0−30 cm from the rubber tree, the RSAD of rubber trees treated with M2 increased by 182.84%, 70.01%, and 133.58% compared to the CK, M1, and M3 treatments. At a distance of 120−150 cm from the rubber tree, the RSAD of banana plants in the M2 treatment was higher than in the M1 and M3 treatments, with increases of 98.06% and 89.45%, respectively.

### 3.3 Underground interspecific competition intensity index

#### 3.3.1 Underground interspecific competition intensity index for each treatment and different soil depths

The root competition dynamics in the rubber tree−banana intercropping system, influenced by various fertilization treatments, exhibited distinct patterns across different soil layers and growth stages of the rubber tree (**Table 1**). During the vigorous growth stage, the highest UICII for the M1 treatment was observed in the 40−60 cm soil layer, while the M2 and M3 treatments peaked in the 20−40 cm soil layer. Notably, the UICII for the M2 treatment was 31.65% and 22.69% lower than that of the M1 and M3 treatments, respectively. In the bud development stage, both M1 and M2 treatments showed their highest UICII values in the 40−60 cm soil layer, while the M3 treatment peaked in the 20−40 cm soil layer, indicating the weakest root competition during this period, with reductions of 5.45% and 4.10% compared to M1 and M2, respectively. By the harvest stage, the highest UICII for all treatments was recorded in the 40−60 cm soil layer, each achieving a value of 1. However, the M3 treatment showed a reduction in root competition by 19.58% and 18.34% compared to M1 and M2 treatments. These results suggest that a 30% substitution of organic nitrogen mitigates root competition across all soil layers during the banana’s vigorous growth stage, while a 45% substitution of slow−release nitrogen effectively reduces root competition during the bud development and harvest stages within the intercropping system.

**Table 1:** Underground interspecific competition intensity index for each treatment and different soil depths.

Growth stage	Soil depth (cm)	Underground interspecific competition intensity index		
		M1	M2	M3
Vigorous growth stage	0-20	0.59	0.54	0.76
	20-40	0.66	0.67	0.90
	40-60	1.00	0.33	0.33
	mean value	0.75aA	0.51aB	0.66aA
Bud development stage	0-20	0.73	0.65	0.89
	20-40	0.87	0.91	0.90
	40-60	1.00	1.00	0.67
	mean value	0.87aA	0.85aA	0.82aA
Harvest stage	0-20	0.82	0.89	0.74
	20-40	0.96	0.85	0.50
	40-60	1.00	1.00	1.00
	mean value	0.93aA	0.91aA	0.75aA

Different lowercase letters indicate significant differences between the treatments in the same stage (*p* < 0.05). Different uppercase letters indicate significant differences of the same treatment between the stages (*p* < 0.05).

#### 3.3.2 Underground interspecific competition intensity index for each treatment and different distance from tree

In terms of interspecific root competition during the banana growth periods, the UICII across various distances from the rubber tree also showed notable differences depending on the fertilization treatment (**Table 2**). During the vigorous growth stage, the UICII for the M1 treatment peaked at a distance of 90 cm from the rubber tree, while the M2 and M3 treatments reached their highest values at 150 cm. In this phase, the UICII of the M2 treatment was 14.91% and 13.14% lower than that of M1 and M3 treatments, respectively. During the banana bud development stage, the highest UICII for both M1 and M2 treatments was observed at 90 cm from the rubber tree, while the M3 treatment peaked at 120 cm. The M2 treatment recorded UICII values that were 23.00% and 22.92% lower than those of M1 and M3 treatments, respectively. By the banana harvest period, the highest UICII for M1 and M3 treatments was found again at 150 cm from the rubber tree, whereas the M2 treatment peaked at 90 cm. Compared to the M1 and M3 treatments, the UICII of the M2 treatment increased by 14.84% and 18.12%, respectively. These results suggest that a 30% substitution of organic nitrogen effectively alleviates root competition intensity during the banana’s vigorous growth and bud development stages while promoting interspecific root competition during the harvest period.

**Table 2:** Underground interspecific competition intensity index for each treatment in different distances from the rubber tree.

Growth stage	Distance from the tree (cm)	Underground interspecific competition intensity index		
		M1	M2	M3
Vigorous growth stage	30	0.11	0.08	0.08
	60	0.28	0.16	0.11
	90	0.30	0.07	0.10
	120	0.26	0.18	0.32
	150	0.00	0.33	0.33
	mean value	0.19aA	0.16aAB	0.19aA
Bud development stage	30	0.22	0.07	0.18
	60	0.19	0.15	0.18
	90	0.37	0.29	0.20
	120	0.00	0.09	0.21
	150	0.00	0.00	0.00
	mean value	0.16aA	0.12aB	0.16aA
Harvest stage	30	0.18	0.14	0.20
	60	0.26	0.25	0.21
	90	0.31	0.45	0.13
	120	0.15	0.25	0.33
	150	0.33	0.33	0.33
	mean value	0.25aA	0.28aA	0.24aA

Different lowercase letters indicate significant differences between the treatments in the same stage (*p*<0.05). Different uppercase letters indicate significant differences of the same treatment between the stages (*p*<0.05).

### 3.4 Soil nutrient

#### 3.4.1 Available N

**Figure 7:**
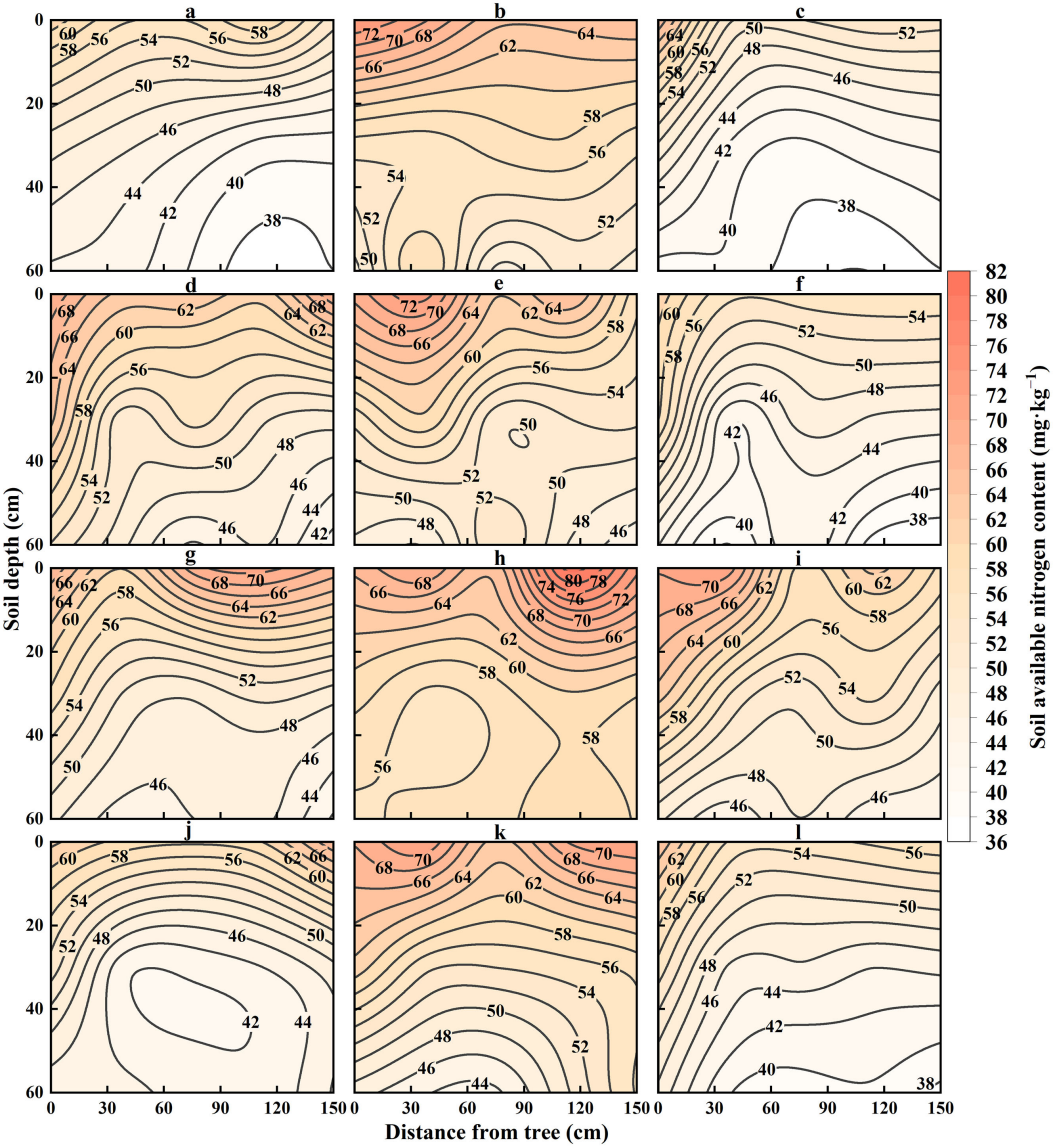
Distribution of soil available nitrogen content. Vigorous growth stage **(A, D, G, F)**, bud development stage **(B, E, H, K)**, harvest stage **(C, F, I, L)**, from top to bottom are CK, M1, M2, M3. CK, control treatment as young rubber tree monoculture; M1, conventional chemical fertilizer treatment; M2, 30% organic nitrogen substitution treatment; M3, controlled released nitrogen fertilizer combined with 45% treatment.

The distribution of soil available nitrogen content in monoculture and intercropped rubber/banana systems under varying fertilization regimes is depicted (**Figure 7**). Overall, soil available nitrogen initially increased and then declined, reaching its peak during the banana bud development stage. Vertically, the highest nitrogen content was observed in the 0−20 cm layer for both monoculture and intercropped systems, with a gradual decrease at greater depths. During the vigorous growth stage (**Figures 7A, D, G, J**), intercropping systems employing the three fertilization treatments resulted in increases in available nitrogen content by 14.49%, 16.93%, and 7.59% in the 0−20 cm layer, 21.57%, 15.41%, and 3.84% in the 20−40 cm layer, and 16.02%, 13.36%, and 10.04% in the 40−60 cm layer, relative to the CK treatment. By the bud development stage (**Figures 7B, E, H, K**), the M2 treatment demonstrated increases of 4.30%, 3.83%, and 3.96% in soil nitrogen content at the 0−20 cm, 20−40 cm, and 40−60 cm depths, respectively, compared to the CK treatment. At the harvest stage (**Figures 7C, F, I, L**), the M1 treatment resulted in increases of 1.60%, 10.91%, and 5.30%, the M2 treatment in increases of 14.43%, 26.49%, and 21.32%, and the M3 treatment in increases of 4.41%, 9.61%, and 7.48% at the 0−20 cm, 20−40 cm, and 40−60 cm layers, respectively, compared to the CK treatment. These results highlight the capacity of the rubber/banana intercropping system to enhance soil nitrogen availability relative to monoculture rubber, with the M2 fertilization mode proving to be the most effective.

#### 3.4.2 Available P

**Figure 8:**
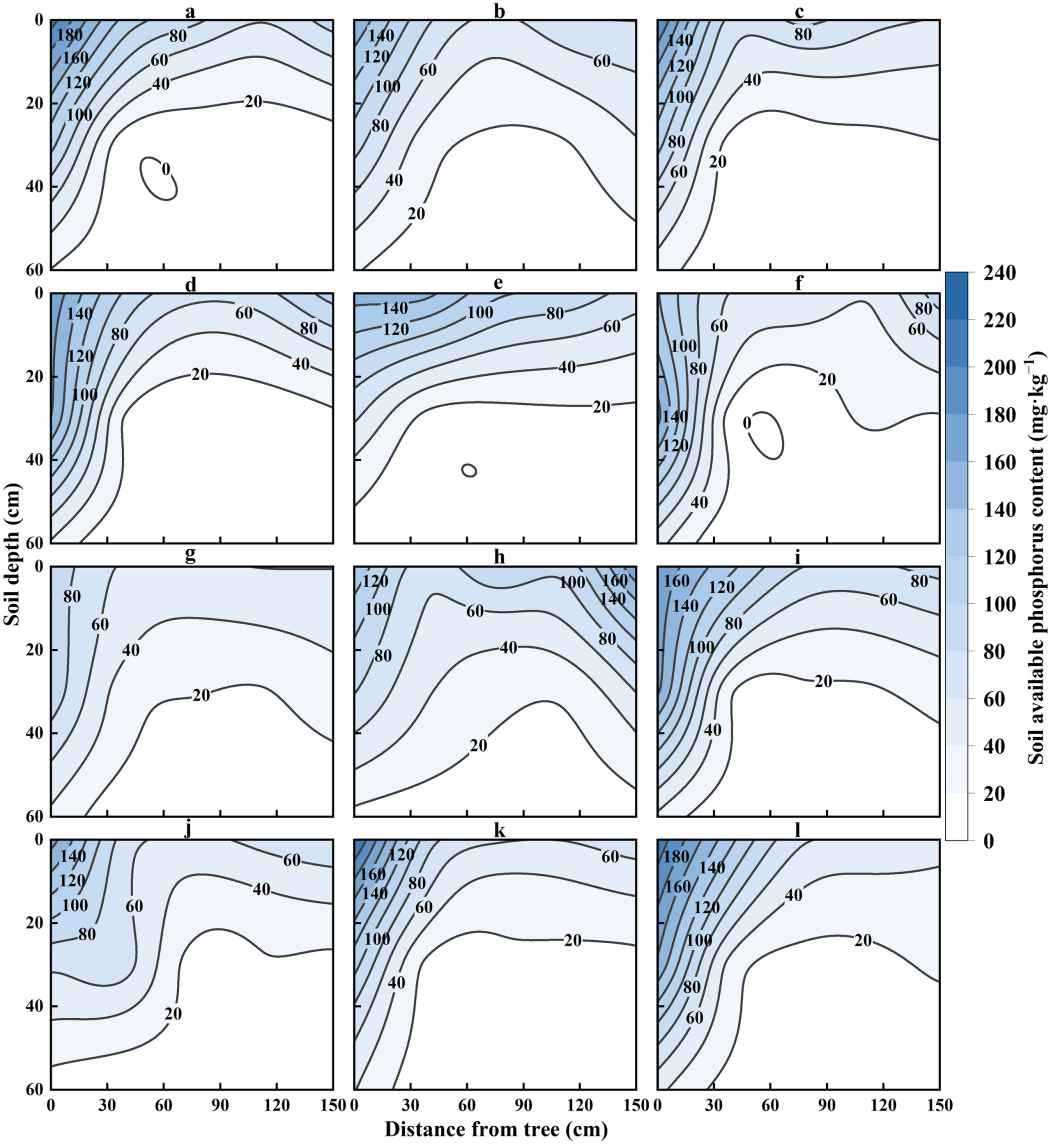
Distribution of soil available phosphorus content. Vigorous growth stage **(A, D, G, F)**, bud development stage **(B, E, H, K)**, harvest stage **(C, F, I, L)**, from top to bottom are CK, M1, M2, M3. CK, control treatment as young rubber tree monoculture; M1, conventional chemical fertilizer treatment; M2, 30% organic nitrogen substitution treatment; M3, controlled released nitrogen fertilizer combined with 45% treatment.

The distribution of soil available phosphorus content in monoculture and intercropped rubber/banana systems under various fertilization modes is presented (**Figure 8**). Generally, the available phosphorus content initially decreased and then increased with increasing distance from the rubber trees. At the vigorous growth stage (**Figures 8A, D, G, J**), the soil available phosphorus content in the 0−20 cm layer was 6.94%, 70.31%, and 28.15% lower in the CK treatment compared to M1, M2, and M3 treatments, respectively. By the bud development stage (**Figures 8B, E, H, K**), available phosphorus content in the 0−20 cm depth increased by 24.12%, 25.89%, and 12.29% in the M1, M2, and M3 treatments, respectively, compared to CK. In terms of horizontal distribution, at the closest distance from the banana, the M2 treatment exhibited significant increases of 119.80%, 149.58%, and 147.97% in the 0−20 cm soil layer relative to CK, M1, and M3 treatments, respectively. At the harvest stage (**Figures 8C, F, I, L**), soil available phosphorus content in the 0−20 cm layer of intercropping systems M2 and M3 treatments increased by 12.72% and 4.80%, respectively, compared to the CK treatment. At a 30 cm distance from the rubber tree, M2 and M3 treatments showed increases of 11.32% and 22.53%, respectively, relative to CK. Overall, at the vigorous growth stage, the M1 treatment increased available phosphorus content by 6.35% compared to CK. During the bud development stage, intercropping treatments M1 to M3 resulted in yield increases ranging from 2.16% to 23.29% compared to CK. At the harvest stage, available phosphorus content in the M2 and M3 treatments rose by 16.66% to 19.45% relative to CK, with the M2 treatment exhibiting the highest level. These results suggest that intercropping treatments generally enhance soil available phosphorus content relative to monocropping, with the 30% organic nitrogen substitution treatment (M2) showing significant advantages during the banana bud development and harvest stages.

#### 3.4.3 Available K

**Figure 9:**
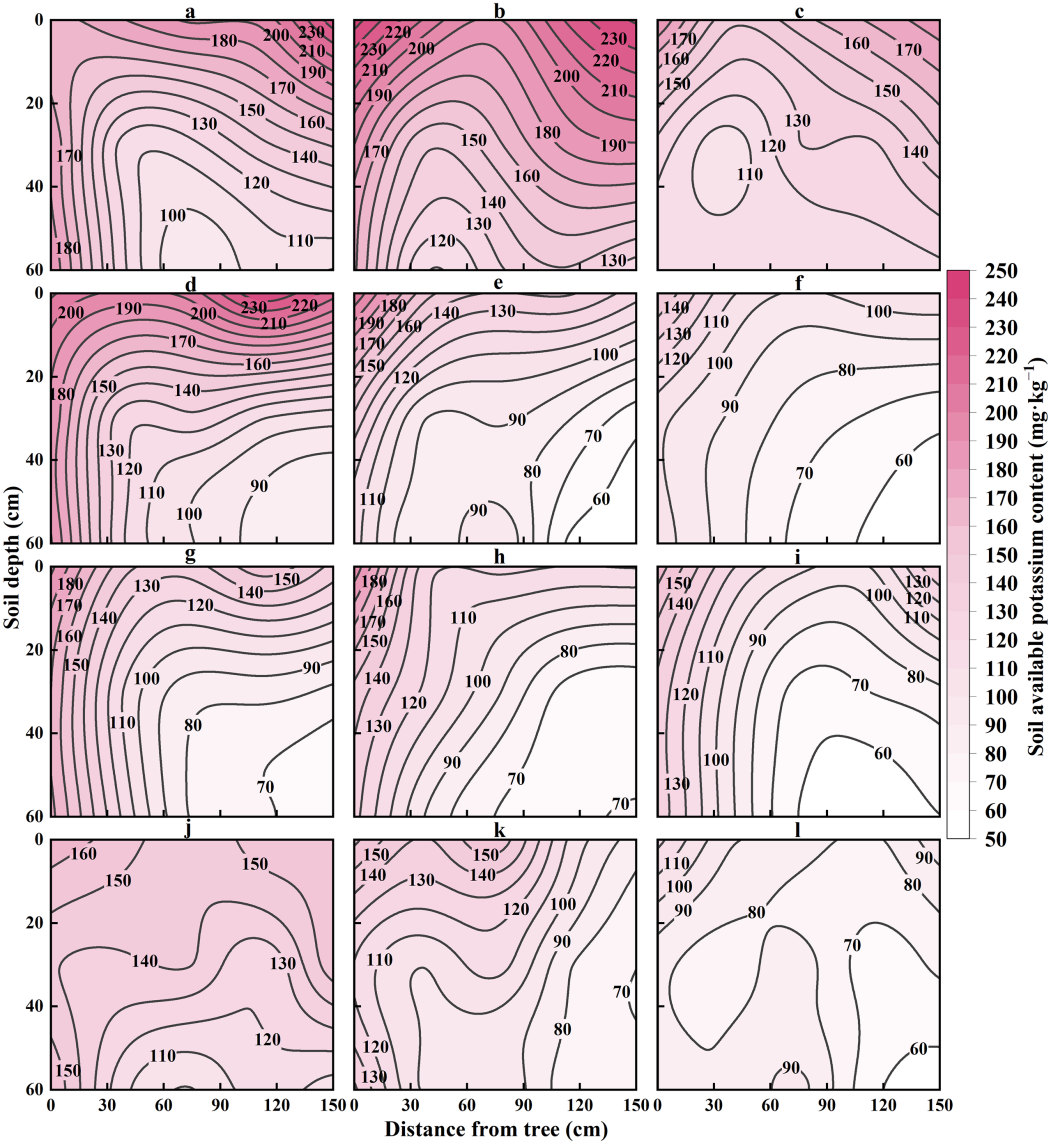
Distribution of soil available potassium content. Vigorous growth stage **(A, D, G, F)**, bud development stage **(B, E, H, K)**, harvest stage **(C, F, I, L)**, from top to bottom are CK, M1, M2, M3. CK, control treatment as young rubber tree monoculture; M1, conventional chemical fertilizer treatment; M2, 30% organic nitrogen substitution treatment; M3, controlled released nitrogen fertilizer combined with 45% treatment.

The distribution of soil available potassium content in monoculture and intercropped rubber/banana systems under three different fertilization regimes is shown (**Figure 9**). Generally, the soil available potassium content in intercropping systems was lower than in monoculture systems. At the vigorous growth stage (**Figures 9A, D, G, J**), in the 0−20 cm soil layer, only the M1 treatment exhibited a 10.17% increase compared to the CK treatment, while the M2 and M3 treatments showed decreases of 26.32% and 25.58%, respectively. At both the bud development stage (**Figures 9B, E, H, K**) and the harvest stage (**Figures 9C, F, I, L**), available potassium content in the 0−20 cm depth decreased by 7.47%−43.82% for M1, 34.05%−60.84% for M2, and 69.02%−78.37% for M3, compared to the CK treatment.

#### 3.4.4 Organic matter

**Figure 10:**
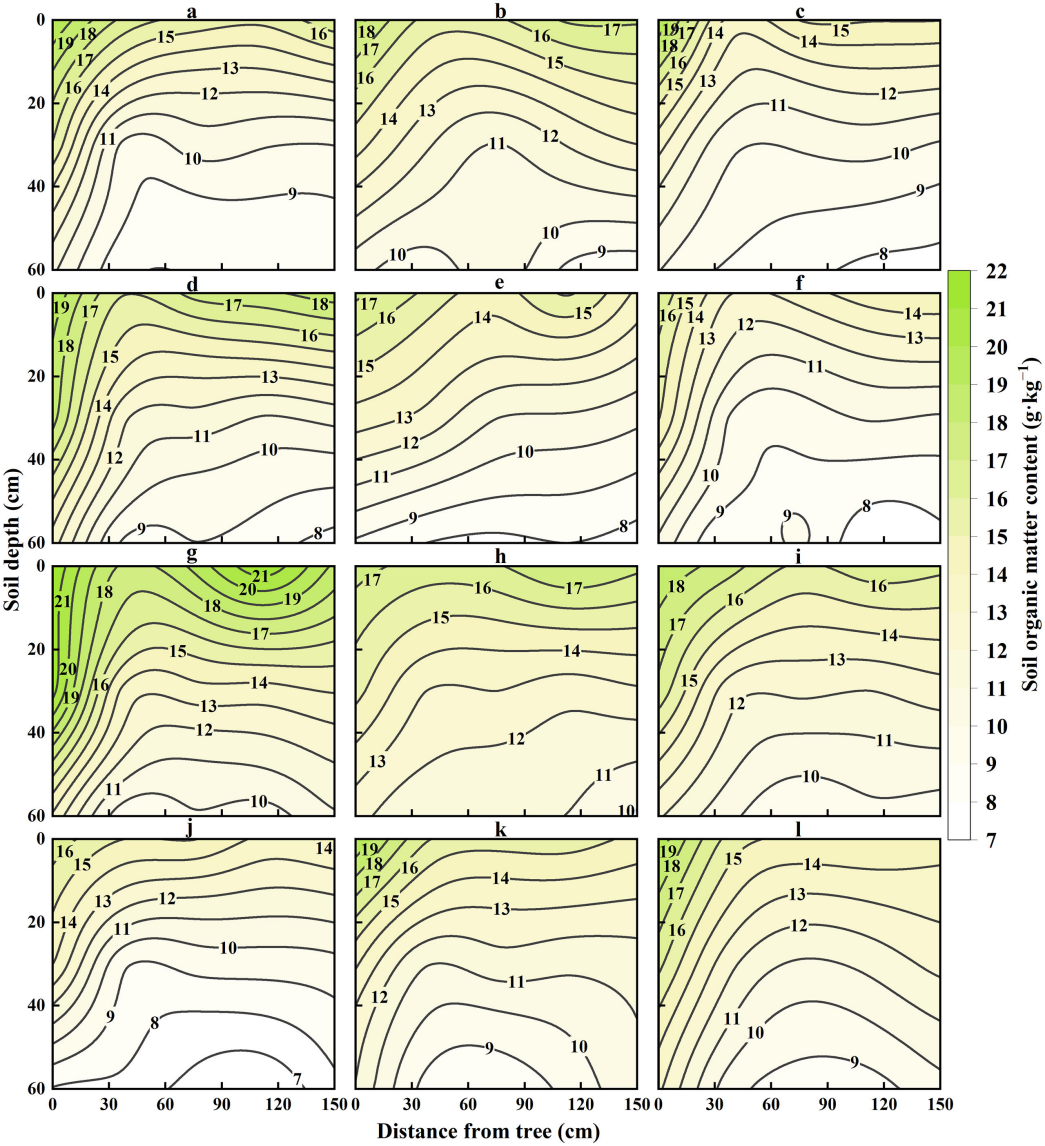
Distribution of soil organic matter content. Vigorous growth stage **(A, D, G, F)**, bud development stage **(B, E, H, K)**, harvest stage **(C, F, I, L)**, from top to bottom are CK, M1, M2, M3. CK, control treatment as young rubber tree monoculture; M1, conventional chemical fertilizer treatment; M2, 30% organic nitrogen substitution treatment; M3, controlled released nitrogen fertilizer combined with 45% treatment.

The distribution of soil organic matter content in monoculture and intercropped rubber/banana systems under three different fertilization regimes is presented (**Figure 10**). Vertically, the highest soil organic matter content was observed in the 0−20 cm layer for both monoculture and intercropping systems. Horizontally, the content was higher at the outer edges and lower at the center. At the vigorous growth stage (**Figures 10A, D, G, J**), soil organic matter content in the 0−20 cm layer increased by 6.22% and 17.71% in the M1 and M2 treatments, respectively, compared to the CK treatment. By the bud development stage (**Figures 10B, E, H, K**), the M2 treatment exhibited the highest soil organic matter content in the 0−20 cm layer, with a 2.34% increase compared to the CK treatment. At the harvest stage (**Figures 10C, F, I, L**), M2 treatment showed a 7.77% increase in soil organic matter content relative to the CK treatment. These results suggest that the M2 treatment effectively enhances soil organic matter content compared to monoculture rubber cultivation.

### 3.5 Response of rhizosphere microbial community structure and diversity to fertilization treatments

#### 3.5.1 Effects of different fertilization treatments on the Alpha diversity of rhizosphere microbial communities

**Figure 11:**
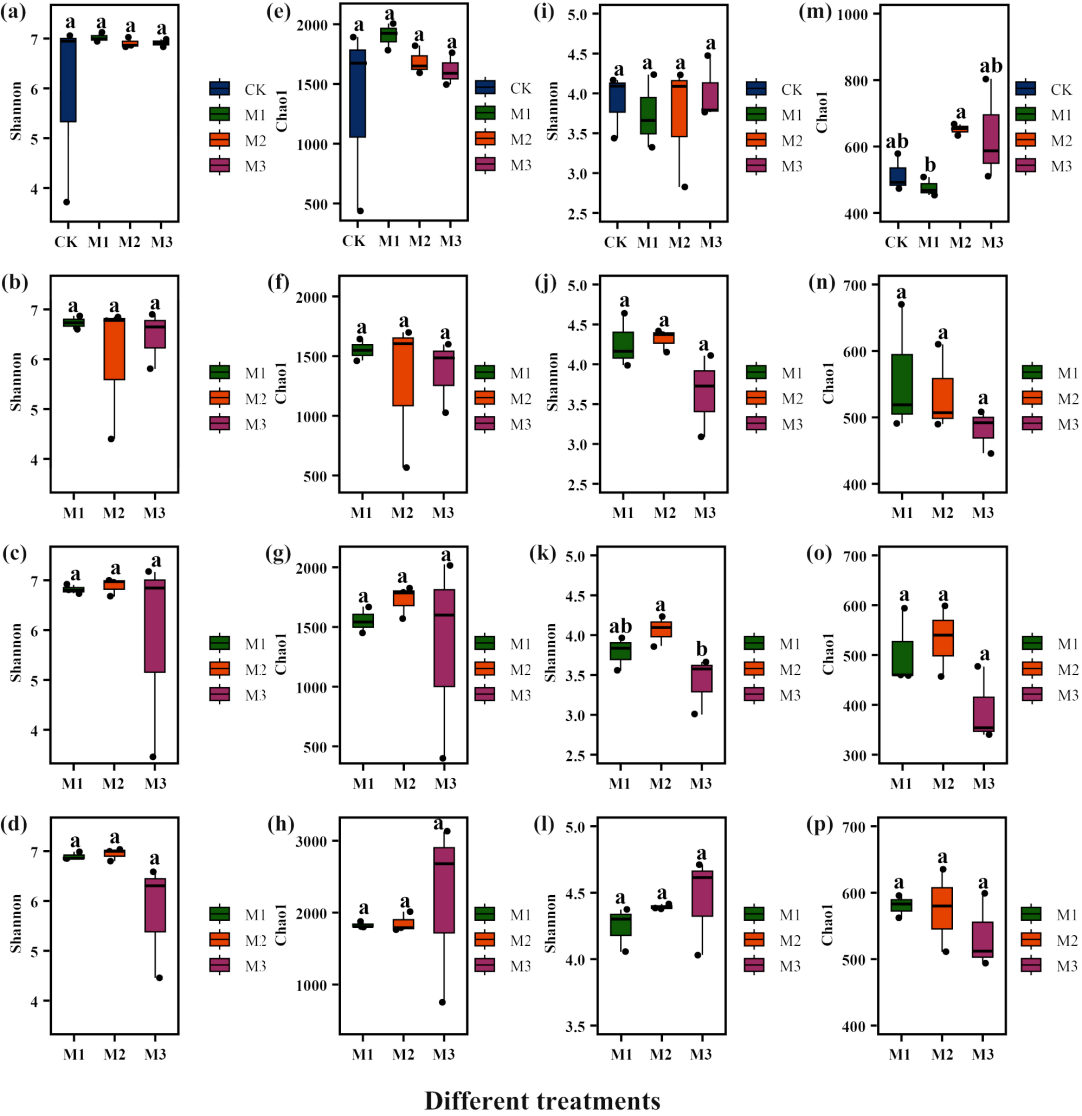
Effects of different fertilization treatments on the alpha diversity of rhizosphere microbial communities in rubber tree and banana at different spatial locations. **(A–D)**: Shannon index of bacterial communities; **(E–H)**: Chao1 index of bacterial communities; **(I–L)**: Shannon index of fungal communities; **(M–P)**: Chao1 index of fungal communities. **(A, E, I, M)**: Rubber tree rhizosphere (0-30 cm from rubber tree); **(B, F, J, N)**: Rubber tree rhizosphere in the interaction zone (60-90 cm from rubber tree); **(C, G, K, O)**: Banana rhizosphere in the interaction zone (60-90 cm from rubber tree); **(D, H, L, P)**: Banana rhizosphere (120-150 cm from rubber tree). CK, control treatment as young rubber tree monoculture; M1, conventional chemical fertilizer treatment; M2, 30% organic nitrogen substitution treatment; M3, controlled released nitrogen fertilizer combined with 45% treatment.

Different fertilization treatments significantly affected the alpha diversity of rhizosphere microbial communities in rubber trees and banana plants (**Figures 11A–P**). Analysis of bacterial communities showed that the Shannon index was highest under the M1 treatment in both the 0–30 cm rubber tree rhizosphere (**Figure 11A**) and the intercropping zone rubber rhizosphere (**Figure 11B**), with values of 7.02 and 6.73, respectively—the former representing an 18.84% increase compared to CK. In contrast, the intercropping zone banana rhizosphere (**Figure 11C**) and the 0–30 cm banana rhizosphere (**Figure 11D**) reached their maximum values under M2, at 6.88 and 6.95, reflecting increases of 18.14% and 20.08% compared to M3, respectively. Chao1 index analysis indicated that the 0–30 cm rubber tree rhizosphere (**Figure 11E**) and intercropping zone rubber rhizosphere (**Figure 11F**) also exhibited the highest values under M1, at 1903.67 and 1551.33, respectively, with the former showing a significant 42.67% increase compared to CK. The intercropping zone banana rhizosphere (**Figure 11G**) reached its maximum under M2 (1726), increasing by 10.93% compared to M1, while the 0–30 cm banana rhizosphere (**Figure 11H**) was highest under M3 (2186.55). Analysis of fungal communities revealed that the Shannon index reached its maximum under M2 in both the intercropping zone rubber rhizosphere (**Figure 11J**) and banana rhizosphere (**Figure 11K**), with values of 4.31 and 4.06, representing increases of 18.50% and 19.03% compared to M3, respectively (the latter being significant at P < 0.05). In contrast, the 0–30 cm rubber tree rhizosphere (**Figure 11I**) and banana rhizosphere (**Figure 11L**) showed the highest values under M3. The Chao1 index indicated that the 0–30 cm rubber tree rhizosphere (**Figure 11M**) and the intercropping zone banana rhizosphere (**Figure 11O**) both reached their maximum under M2, with values of 651.67 and 532, reflecting significant increases of 36.71% (*p* < 0.05) and 36.29% compared to the control treatment, respectively. The intercropping zone rubber rhizosphere (**Figure 11N**) and 0–30 cm banana rhizosphere (**Figure 11P**), however, showed the highest values under M1. These results indicate that the M2 treatment has a significant advantage in enhancing bacterial diversity in the banana rhizosphere and fungal community richness in the intercropping zone, suggesting that this fertilization approach plays a positive role in improving the rhizosphere micro−ecological environment.

#### 3.5.2 Effects of different fertilization treatments on the rhizosphere bacterial community characteristics of rubber trees and banana plants

**Figure 12:**
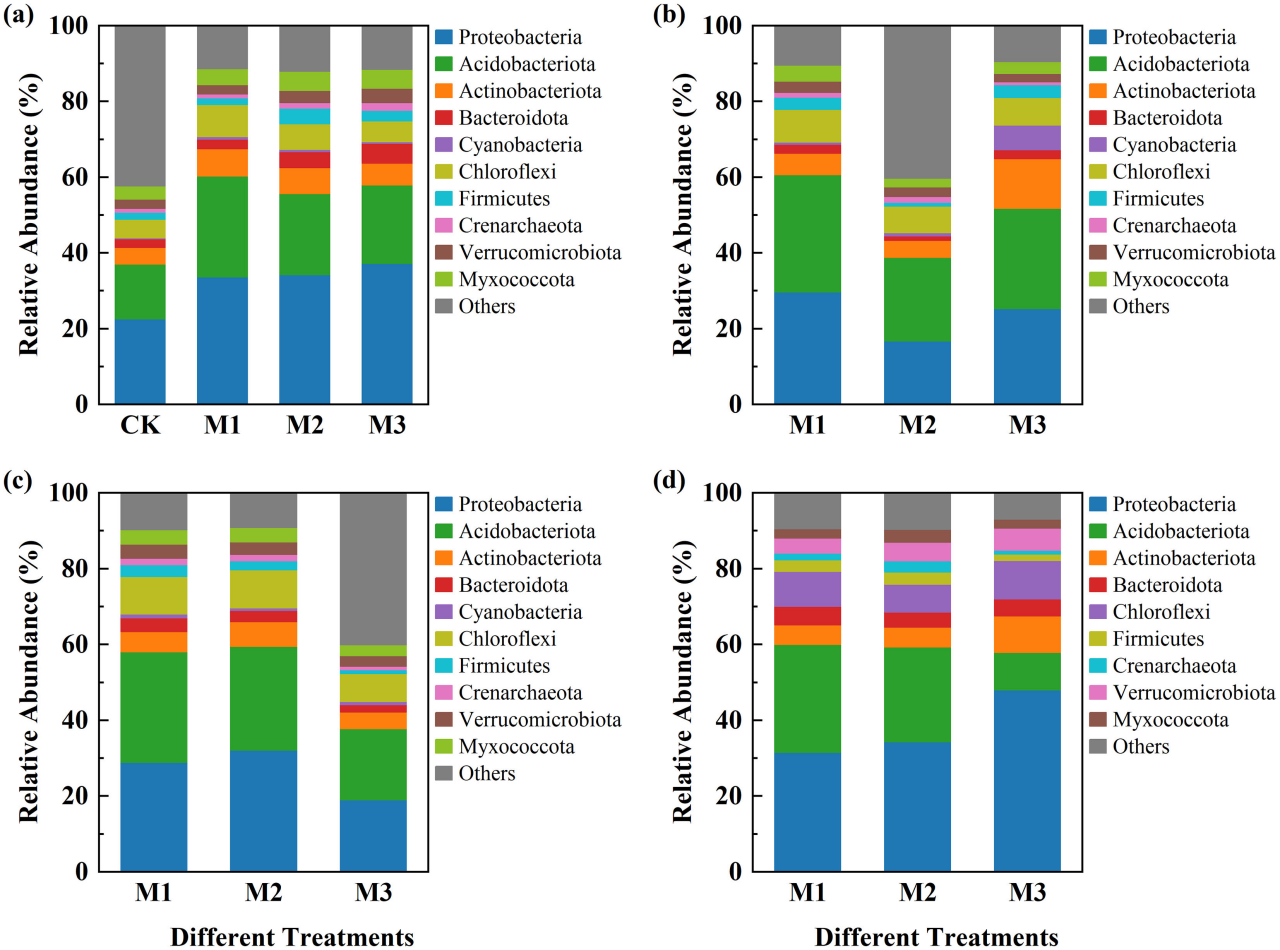
Effects of different fertilization treatments on the composition of rhizosphere bacterial communities in rubber tree and banana at different spatial locations. **(A)**: Rubber tree rhizosphere (0-30 cm from rubber tree); **(B)**: Rubber tree rhizosphere in the interaction zone (60-90 cm from rubber tree); **(C)**: Banana rhizosphere in the interaction zone (60-90 cm from rubber tree); **(D)**: Banana rhizosphere (120-150 cm from rubber tree). CK, control treatment as young rubber tree monoculture; M1, conventional chemical fertilizer treatment; M2, 30% organic nitrogen substitution treatment; M3, controlled released nitrogen fertilizer combined with 45% treatment.

Different fertilization treatments significantly altered the rhizosphere bacterial community structure of both rubber trees and banana plants (**Figures 12A–D**), with the M2 treatment showing a notable advantage in increasing the relative abundance of multiple beneficial bacterial phyla. In the rubber tree rhizosphere (**Figure 12A**), M2 significantly increased the relative abundance of Bacteroidota and Firmicutes, with Bacteroidota increasing by 66.40% compared to M1 (*p* < 0.05) and Firmicutes increasing by 131.00% and 45.58% compared to M1 and M3, respectively. In the intercropping zone rubber tree rhizosphere (**Figure 12B**), Crenarchaeota reached its highest relative abundance under M2, increasing by 24.00% and 107.00% compared to M1 and M3. The intercropping zone banana rhizosphere (**Figure 12C**) exhibited particularly pronounced responses under M2, where Proteobacteria increased by 11.26% and 68.61% compared to M1 and M3, Actinobacteria increased by 20.96% and 49.54%, and Chloroflexi increased by 37.09% compared to M3. In the banana rhizosphere (**Figure 12D**), M2 also significantly enhanced the relative abundance of several phyla: Acidobacteriota increased by 152.00% compared to M3 (*p* < 0.05), Firmicutes increased by 92.54% compared to M3, Crenarchaeota increased by 69.84% compared to M1, and Myxococcota increased by 37.70% and 43.37% compared to M1 and M3. In summary, the M2 treatment demonstrated a significant advantage in promoting the assembly of beneficial rhizosphere bacterial communities.

#### 3.5.3 Effects of different fertilization treatments on the rhizosphere fungal community characteristics of rubber trees and banana plants

**Figure 13:**
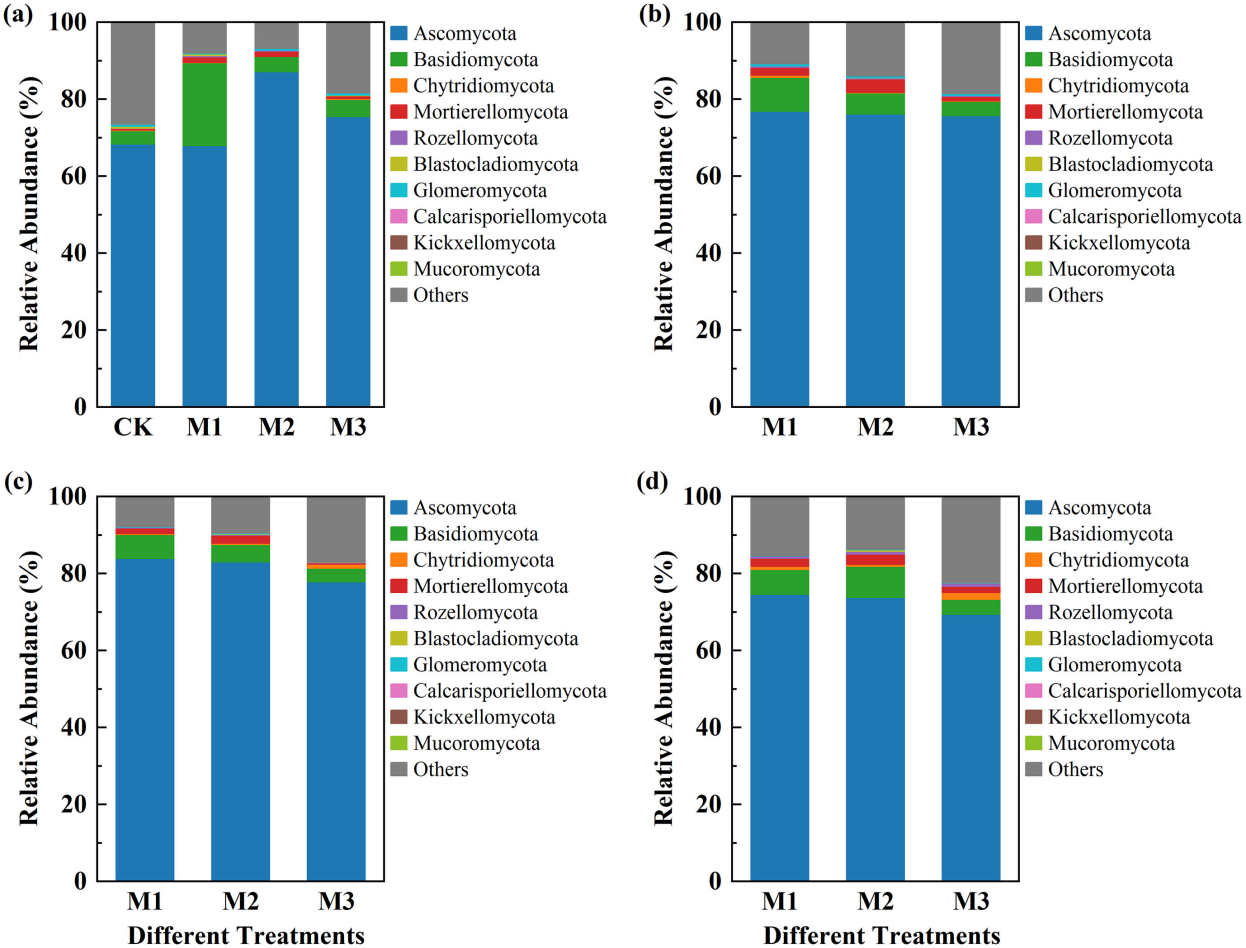
Effects of different fertilization treatments on the composition of rhizosphere fungal communities in rubber tree and banana at different spatial locations. **(A)**: Rubber tree rhizosphere (0-30 cm from rubber tree); **(B)**: Rubber tree rhizosphere in the interaction zone (60-90 cm from rubber tree); **(C)**: Banana rhizosphere in the interaction zone (60-90 cm from rubber tree); **(D)**: Banana rhizosphere (120-150 cm from rubber tree). CK, control treatment as young rubber tree monoculture; M1, conventional chemical fertilizer treatment; M2, 30% organic nitrogen substitution treatment; M3, controlled released nitrogen fertilizer combined with 45% treatment.

Different fertilization treatments significantly influenced the rhizosphere fungal community structure of rubber trees and banana plants at the phylum level (**Figures 13A–D**), with the M2 treatment demonstrating notable advantages in increasing the relative abundance of several key fungal phyla. In the rubber tree rhizosphere (**Figure 13A**), the relative abundance of Ascomycota under M2 reached 87.15%, representing a 28.36% increase compared to M1, while Basidiomycota was highest under M1 (21.58%). In the intercropping zone rubber tree rhizosphere (**Figure 13B**), Mortierellomycota showed the highest relative abundance under M2 (3.51%), with a significant increase of 205.00% compared to M3 (*p* < 0.05). In the intercropping zone banana rhizosphere (**Figure 13C**), the relative abundance of Mortierellomycota under M2 was 2.07%, reflecting a 38.08% increase compared to M1. In the banana rhizosphere (**Figure 13D**), all observed phyla performed optimally under M2: the relative abundance of Ascomycota was 73.76%, increasing by 6.31% compared to M3; the relative abundance of Basidiomycota was 8.11%, with significant increases of 24.62% and 111.00% compared to M1 and M3, respectively; and the relative abundance of Mortierellomycota was 2.69%, increasing by 27.42% and 65.53% compared to M1 and M3. In summary, the M2 treatment exhibited a clear advantage in promoting the establishment of beneficial rhizosphere fungal communities.

### 3.6 Interactions between rhizosphere microbial communities and root−soil system factors

#### 3.6.1 Correlation analysis between bacterial communities and root/soil factors

**Figure 14:**
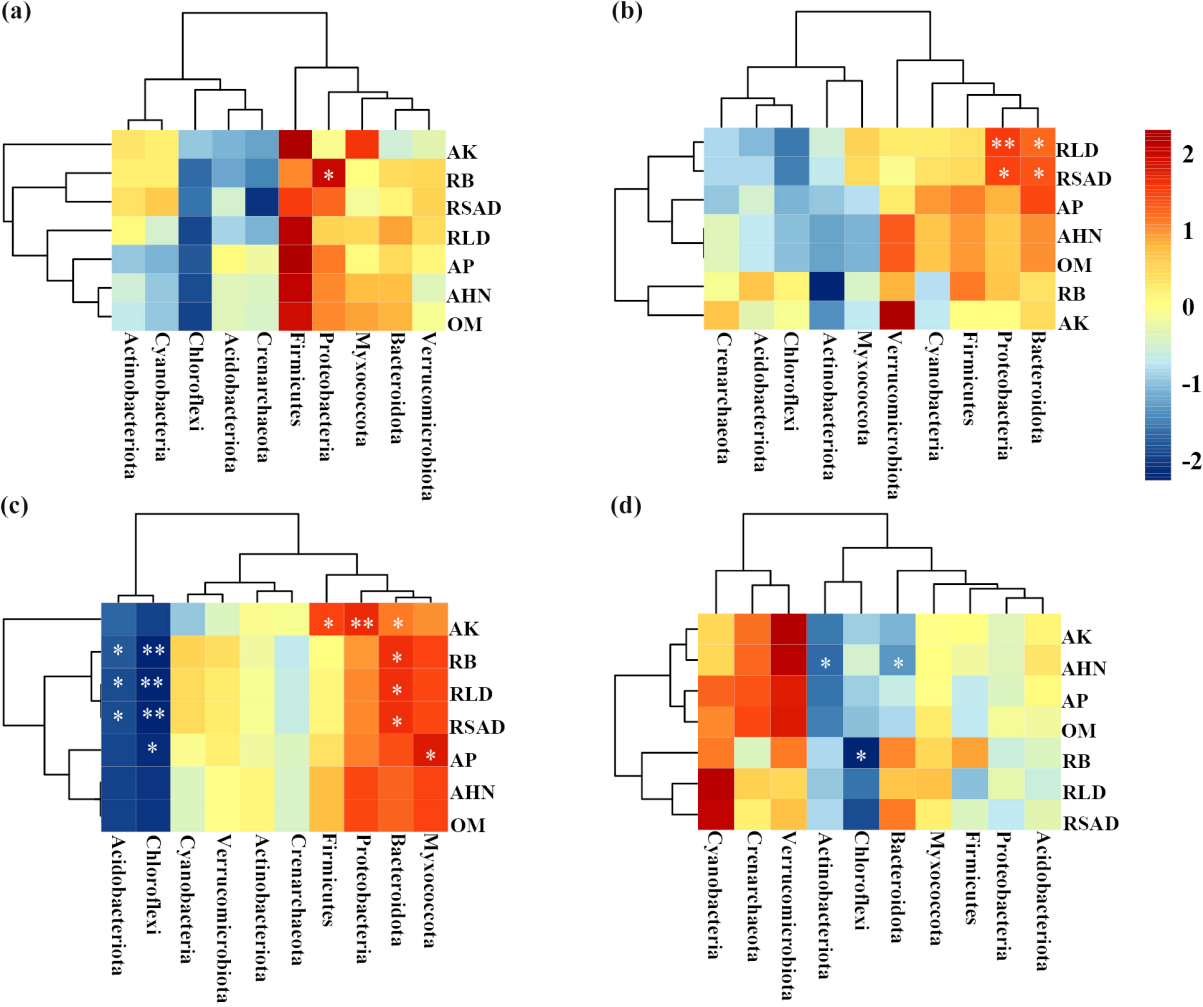
Correlation analysis between rhizosphere bacterial communities and root/soil factors under different fertilization treatments. **(a)** Rubber tree rhizosphere (0 -30 cm from rubber tree); **(b)** Rubber tree rhizosphere in the interaction zone (60 -90 cm from rubber tree); **(c)** Banana rhizosphere in the interaction zone (60 -90 cm from rubber tree); **(d)** Banana rhizosphere (120 -150 cm from rubber tree). CK, control treatment as young rubber tree monoculture; M1, conventional chemical fertilizer treatment; M2, 30% organic nitrogen substitution treatment; M3, controlled released nitrogen fertilizer combined with 45% treatment. * indicates statistical significance at p<0.05, and ** indicates p<0.01.

Correlation analysis between rhizosphere bacterial communities and root indices as well as soil factors under different fertilization treatments is shown in **Figure 14**. In the 0–30 cm rubber tree rhizosphere (**Figure 14A**), the relative abundance of Proteobacteria showed a significant positive correlation with root biomass (*p* < 0.05). In the intercropping zone rubber tree rhizosphere (**Figure 14B**), Bacteroidota was significantly positively correlated with root length density and root surface area density, while Proteobacteria was highly significantly positively correlated with root length density (p < 0.01) and significantly positively correlated with root surface area density. Analysis in the intercropping zone banana rhizosphere (**Figure 14C**) indicated that Acidobacteriota and Chloroflexi were significantly negatively correlated with root biomass, root length density, and root surface area density, with Chloroflexi showing highly significant negative correlations with these root indices and a significant negative correlation with soil available phosphorus. Firmicutes was significantly positively correlated with soil available potassium, while *Proteobacteria* was highly significantly positively correlated with available potassium. Bacteroidota showed significant positive correlations with available potassium, root biomass, root length density, and root surface area density, and Myxococcota was significantly positively correlated with soil available phosphorus. In the 0–30 cm banana rhizosphere (**Figure 14D**), Actinobacteria and Bacteroidota were significantly negatively correlated with soil alkaline hydrolyzable nitrogen, and Chloroflexi was significantly negatively correlated with root biomass. It is worth noting that beneficial bacterial groups such as Proteobacteria and Bacteroidota, which were significantly enriched in the M2 treatment, exhibited significant positive correlations with both root development indices and soil nutrient availability, revealing the potential mechanism by which this fertilization approach promotes plant growth from a microbial ecological perspective.

#### 3.6.2 Correlation analysis between fungal communities and root/soil factors

**Figure 15:**
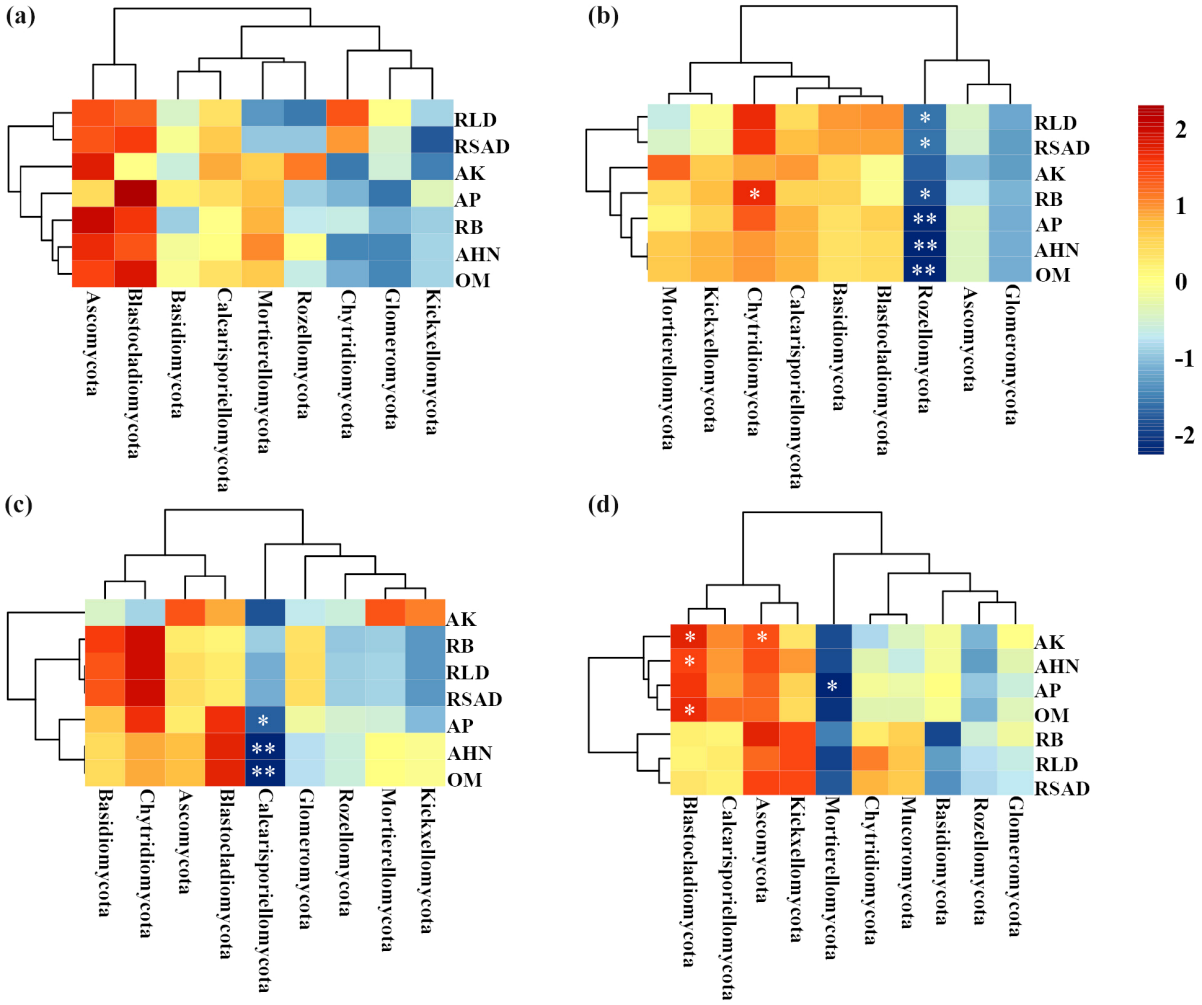
Correlation analysis between rhizosphere fungal communities and root/soil factors under different fertilization treatments. **(A)**: Rubber tree rhizosphere (0-30 cm from rubber tree); **(B)**: Rubber tree rhizosphere in the interaction zone (60-90 cm from rubber tree); **(C)**: Banana rhizosphere in the interaction zone (60-90 cm from rubber tree); **(D)**: Banana rhizosphere (120-150 cm from rubber tree). CK, control treatment as young rubber tree monoculture; M1, conventional chemical fertilizer treatment; M2, 30% organic nitrogen substitution treatment; M3, controlled released nitrogen fertilizer combined with 45% treatment.

Correlation analysis between fungal community structure at the phylum level and root indices as well as soil factors is shown in **Figure 15**. In the 0−30 cm rubber tree rhizosphere (**Figure 15A**), no significant correlations were observed between any fungal phyla and the root or environmental factors. Analysis in the intercropping zone rubber tree rhizosphere (**Figure 15B**) indicated that Chytridiomycota showed a significant positive correlation with root biomass, whereas Rozellomycota was significantly negatively correlated with root biomass, root length density, and root surface area density, and exhibited highly significant negative correlations with soil available phosphorus, alkaline hydrolyzable nitrogen, and organic matter content. In the intercropping zone banana rhizosphere (**Figure 15C**), the relative abundance of Calcarisporiellomycota was significantly negatively correlated with soil available phosphorus and highly significantly negatively correlated with alkaline hydrolyzable nitrogen and organic matter. Analysis in the 0−30 cm banana rhizosphere (**Figure 15D**) revealed that Blastocladiomycota was significantly positively correlated with soil available potassium, alkaline hydrolyzable nitrogen, and organic matter; Ascomycota was significantly positively correlated with available potassium; and Mortierellomycota was significantly negatively correlated with available phosphorus. It is worth noting that fungal taxa such as Blastocladiomycota, which were significantly enriched in the M2 treatment, showed significant positive correlations with soil nutrient availability, indicating that this fertilization approach may improve soil fertility by regulating the structure of fungal communities.

## 4 Discussion

### 4.1 Roots morphology in intercropping

Competition for underground resources in agroforestry systems is influenced by the distribution and density of root systems (Noordwijk et al., 2015). However, research on the root system characteristics of rubber trees intercropped with other crops under varying fertilization regimes remains limited. A study on nitrogen fertilization levels for intercropped rubber trees and calla lilies revealed that intercropping only inhibited calla lily growth when nitrogen application was below 200 kg·ha^−1^, while it enhanced root system development by increasing root number and length and promoting dry matter allocation to the root system (Li and Lin, 2021). In the present study, during the banana bud development stage, both rubber tree and banana root biomass showed increases at various soil depths under the M2 treatment compared to other treatments. Specifically, within the 0–30 cm range from the rubber tree, root biomass in the M2 treatment was 7.09% to 29.17% higher than in the other treatments (**Figures 4B, E, H, K**). Furthermore, during the banana harvest period, monoculture rubber trees exhibited superior RLD compared to the intercropping treatments. Analysis of vertical spatial changes revealed that, during the budding stage, the total banana RLD in the 0–20 cm soil layer increased by 88.50% and 79.98% under the M2 treatment compared to the M1 and M3 treatments, respectively (**Figures 5F, I, L**). Additionally, within the 0–30 cm range from the rubber tree during the bud development stage, rubber tree RLD under the M2 treatment exceeded that of the CK, M1, and M3 treatments, with increases of 203.37%, 34.53%, and 124.02%, respectively (**Figures 5B, E, H, K**). Moreover, during the banana harvest period, the RSAD was highest under monoculture rubber trees, with a value of 0.116 cm^2^·cm^−3^ (**Figures 6C, F, I, L**). These findings indicate that the temporal and spatial distribution of root system morphology is influenced by growth stages, underscoring the benefits of both monoculture and intercropping systems. The M2 fertilization regime in rubber/banana intercropping systems plays a pivotal role in optimizing root system competition and spatial niche adaptation, demonstrating significant advantages in complementarity.

Research on intercropping rubber trees with other crops has demonstrated that in deeper soil layers, the fine RLD of rubber trees intercropped with kudzu reaches 0.92 cm·cm^−3^, with a biomass density of 0.74 g·dm^−3^. This represents a significant increase of 26.03% and 89.74%, respectively, compared to monoculture (Clermont−Dauphin et al., 2018). Similarly, rubber trees in agroforestry systems intercropped with perennial galangal, tea, and cocoa exhibited average root length densities of 0.18 cm·cm^−3^, 0.18 cm·cm^−3^, and 0.13 cm·cm^−3^, respectively, indicating varying degrees of decline compared to monoculture. A substantial portion of fine rubber tree roots (60.7%–72.5%) were concentrated in the 0–20 cm soil layer across all treatments (Yang et al., 2020). Studies on other intercrops reveal that intercropped corn and soybeans exhibit significantly greater RLD and RSAD compared to monoculture, while intercropped peanuts experience restricted root systems with lower densities (Zheng et al., 2022a). Intercropping wheat and corn resulted in a reduction in root biomass density, RLD, and RSAD of intercropped corn by 53%, 81%, and 70%, respectively, compared to monocrop corn (Wang et al., 2018). Furthermore, intercropping walnuts and wheat led to a significant decrease in RLD, RSAD, and other root morphological indicators compared to monoculture (Duan et al., 2017). Overall, this study demonstrates that the treatment involving 30% organic nitrogen substitution (M2) yielded favorable outcomes in terms of root morphology plasticity and spatial distribution within the young rubber tree/banana intercropping system. It appears that competition for underground resources between trees and crops in agroforestry systems is influenced by root distribution and spatial allocation (Forey et al., 2017). By strategically organizing root distribution within the system, competition can be minimized, thereby enhancing the productivity of the entire agroforestry system (Li et al., 2021; Wei et al., 2024).

We acknowledge that root diameter and specific root length (SRL) were not measured in this study; these traits are valuable for interpreting resource acquisition strategies. Future investigations should incorporate them to further elucidate the physiological basis of the root distribution patterns observed here.

### 4.2 Underground interspecific competition intensity index in intercropping systems

Crop roots compete for nutrient resources in the soil, and when the demand for these resources exceeds their availability, interspecific competition is inevitable (Wang et al., 2018). The nature of this competition—whether complementary or competitive—depends largely on the distribution and density of root systems (Noordwijk et al., 2015). Additionally, the dynamics of root competition are influenced by the spatial arrangement of crops. For instance, when crops are planted in close proximity, their roots are likely to compete more intensely for the same volume of soil, potentially reducing nutrient uptake for both species. In contrast, when root systems are differentiated by depth and spread, they can access distinct soil layers, thereby reducing competition and improving overall resource utilization efficiency (Liu et al., 2022).

We interpret UICII as an indicator of root spatial niche overlap, which may imply potential competition, but actual competitive outcomes also depend on root physiological activity and soil nutrient supply. In this study, the M3 treatment exhibited a lower root competition intensity index (UICII) during the harvest stage compared to the bud development stage, reflecting a reduction in vertical root competition. In contrast, UICII values for both the M1 and M2 treatments increased as rubber trees and bananas progressed through their growth stages. Specifically, the M2 treatment showed a lower UICII during the vigorous growth stage, whereas the M3 treatment recorded the lowest values during both the bud development and harvest stages (**Table 1**). In terms of horizontal root competition, the M2 treatment demonstrated lower UICII values during the vigorous growth and bud development stages, while the M3 treatment exhibited a lower index during the harvest stage. This is likely attributed to the slower nutrient release and prolonged efficacy of organic and controlled−release fertilizers (**Table 2**). The 30% organic fertilizer substitution combined with 45% controlled−release nitrogen significantly reduced root competition throughout the banana growth cycle, thereby improving ecological niche complementarity. Previous research has shown that excessive root competition in agroforestry systems exacerbates resource competition, leading to shortages that hinder efficient resource utilization in intercropping systems (Dai et al., 2021; Liu et al., 2019). Moreover, interspecific competition within such systems affects the root growth of fruit trees, with crops competing for essential resources, ultimately influencing system−wide yields (Zhao et al., 2022). For example, the concentration of roots from multiple crops can intensify belowground competition, potentially reducing crop yields (Zhang et al., 2013). This highlights that the advantages of intercropping extend beyond mere resource competition and include complex root interactions that can enhance plant health and productivity. Root competition in intercropping systems presents a multifaceted challenge, influenced by factors such as root structure and spatial arrangement. A comprehensive understanding of these factors is critical for optimizing intercropping practices to improve crop yields and bolster the sustainability of agricultural systems. Effective agronomic interventions, such as rational fertilization, are essential for mitigating the negative impacts of root competition.

### 4.3 Soil nutrients in intercropping

Intercropping enhances soil fertility, improves soil quality, and optimizes nutrient utilization (Duan et al., 2019; Li et al., 2021). Previous studies have demonstrated that intercropping systems can increase the availability of essential macronutrients—nitrogen, phosphorus, and potassium—which are critical for plant growth (Shen et al., 2024). The current study reveals that, compared to rubber monoculture, the rubber/banana intercropping system significantly increased soil nitrogen availability, with the M2 fertilization treatment proving most effective (**Figure 7**). Regarding soil phosphorus content, the M1 treatment showed certain advantages over the CK treatment during the vigorous growth stage. In both the bud development and harvest stages, the M2 treatment outperformed monoculture and other intercropping fertilization treatments (**Figure 8**). Additionally, the M2 treatment consistently exhibited superior outcomes in soil organic matter content, reaching its peak value across various banana growth stages (**Figure 10**). In contrast, intercropping generally led to a decrease in soil potassium content (**Figure 9**). Previous research has indicated that transitioning rubber trees from monoculture to agroforestry systems resulted in significant increases in soil nitrogen and phosphorus by 38.5% and 48.2%, respectively, suggesting that rubber tree intercropping improves the physical and chemical properties of soil, thereby contributing to the sustainable development of agriculture and the environment (Chen et al., 2019). Multiple studies have reported that intercropping enhances soil organic matter content (Cong et al., 2015; Li et al., 2021; Wei et al., 2024). However, studies on rubber tree and calla lily intercropping indicate that such practices may reduce soil potassium levels (Li and Lin, 2021). Organic fertilizers, rich in nutrients and organic matter, have been shown to significantly improve soil fertility and fertilizer efficiency (Ma et al., 2022; Saudy et al., 2020), which may explain the observed relationship between soil available nitrogen, phosphorus, and the M2 treatment in this study. The higher organic matter content observed in this study, compared to monoculture and other fertilization treatments, is likely attributed to these factors.

Beyond its agronomic benefits, the practical adoption of M2 also depends on the availability and cost of organic fertilizers. In Hainan, organic resources such as manure and compost are relatively accessible, but labor costs for application may be higher than for chemical fertilizers. To improve feasibility, we suggest integrating organic fertilizer with fertigation or using pelletized products; however, a full cost−benefit analysis over the entire rotation cycle is needed to confirm economic viability. In conclusion, intercropping is a sustainable agricultural practice that integrates diverse plant species, enabling optimized nutrient cycling, enhanced soil health, and increased crop yields. This approach is therefore a valuable strategy for sustainable agriculture, offering benefits beyond nutrient acquisition, such as long−term improvements in soil fertility and ecosystem health.

### 4.4 Fertilization effects on rhizosphere microbial communities

This study demonstrated that the M2 treatment (combined organic−inorganic fertilization) most effectively enhanced the rhizosphere microbial community structure and diversity in the rubber tree−banana intercropping system. For bacteria, M2 significantly increased the relative abundance of beneficial phyla such as Bacteroidota and Firmicutes in the rubber tree rhizosphere, and Proteobacteria, Actinobacteria, and Chloroflexi in the intercropped banana rhizosphere (**Figure 11**), which are pivotal for nutrient cycling and soil health (Liu et al., 2020; Ning et al., 2020). Notable increases in Acidobacteriota and Myxococcota under M2 further highlighted the role of organic inputs in shaping microbial assembly (Liu et al., 2020; Yang et al., 2024). Concurrently, M2 enhanced the fungal community by boosting the relative abundance of Ascomycota, Basidiomycota, and Mortierellomycota (**Figure 12**), taxa essential for organic matter decomposition and pathogen suppression (Jianhong et al., 2021; Zhao et al., 2024). The significant enrichment of Mortierellomycota suggests a role in phosphorus solubilization, supported by findings that organic fertilizers stimulate P−cycling microbes (Asif et al., 2023; Zhu et al., 2024). The enhanced bacterial alpha diversity in the banana rhizosphere under M2 (**Figures 13C, D**) confirms that this fertilization approach fosters a more stable and functionally diverse rhizosphere micro−ecology, resonating with the benefits of integrated NPKM practices observed in other systems (Asif et al., 2023; Xu et al., 2017).

The positive influence of the M2 treatment on plant−microbe−soil interactions was further elucidated through correlation analysis. Key bacterial taxa enriched by M2, specifically Proteobacteria and Bacteroidota, showed significant positive correlations with root development indices (root length density, root surface area) and soil available potassium (**Figure 14**), indicating their potential role in promoting banana growth and K utilization. Similarly, the fungal phylum Blastocladiomycota, which was positively correlated with soil nutrients (**Figure 15**), suggests a mechanism through which M2 improves soil fertility by regulating the fungal community (Guo et al., 2019; Yang et al., 2024). In summary, the M2 treatment optimized both the composition and diversity of bacterial and fungal communities, enhancing the efficiency of plant−soil system interactions. This aligns with conclusions from long−term experiments that advocate the “manure + chemical fertilizer” model to enhance microbial functions and crop productivity (Asif et al., 2023; Ren et al., 2021; Xu et al., 2017). Future research should integrate metagenomics and metabolomics to unravel the precise functional mechanisms of these key microbial taxa, thereby informing the design of precision fertilization strategies for sustainable intercropping systems (Chen et al., 2021; Zhao et al., 2024; Zou et al., 2024). It should be noted that our findings are derived from the first year of a young intercropping system. Long−term responses of root interactions, soil nutrient dynamics, and microbial succession under organic substitution may differ as rubber trees mature and their root systems expand. Therefore, multi−year field trials are warranted to confirm the durability of the observed benefits.

## 5 Conclusion

The intercropping system of young rubber trees and bananas, under various fertilization regimes, significantly impacts the temporal and spatial distribution of their root systems, interspecific competition, soil nutrient availability, and rhizosphere microbial communities. Notably, under our experimental conditions, the strategy of replacing 30% of chemical nitrogen with organic fertilizer (the M2 treatment) appears promising under the experimental conditions of this study. This approach alleviates root competition and ecological challenges across most growth stages while enhancing complementary effects and improving soil nitrogen, phosphorus, and organic matter content. Critically, the M2 treatment was most effective in modulating the rhizosphere micro−ecological environment, fostering a more beneficial microbial community structure and enhancing bacterial diversity in the banana rhizosphere. The observed positive correlations between these optimized microbial communities, root development indices, and soil nutrient availability underscore the role of microbial regulation in the improved plant growth and system performance under the M2 regimen. The findings of this study provide preliminary theoretical and practical insights for optimizing fertilization strategies in young rubber−banana intercropping systems in the studied tropical environment (Hainan, China). Furthermore, these results lay the groundwork for future investigations including long−term field trials across different soil types and climates to validate the durability and broader applicability of the observed benefits. Future research should also focus on unraveling the molecular mechanisms that underpin the advantages of the rubber−banana intercropping system under this fertilization framework, particularly the functional roles of key microbial taxa in facilitating nutrient cycling and plant health. Overall, this study provides a scientifically−grounded and potentially sustainable management strategy for enhancing the productivity and ecological sustainability of rubber tree−banana intercropping systems.

## Data Availability

The raw sequence data generated in this study have been deposited in the NCBI Sequence Read Archive (SRA) under BioProject accession number PRJNA1497362.
