# Supplementary material for: Optimizing belowground interactions in young rubber-banana intercropping via partial organic fertilizer substitution: root competition, soil nutrients, and microbial communities
Source: Front Plant Sci. 2026 Jul 31;17:1896674. doi: 10.3389/fpls.2026.1896674 (PMC13473379; doi:10.3389/fpls.2026.1896674)
Supplement: Supplementary file 1 [file Table1.docx]

**Table S1**

Specific quantities of fertilizer for bananas.

| Treatment | Growth stage | Amount per plant (g·plant^-1^) | | | | | | Fertilization times |
| --- | --- | --- | --- | --- | --- | --- | --- | --- |
|  |  | Diammonium phosphate | Urea | Potassium chloride | Shelf-coated urea | Organic fertilizer | Organic water-soluble fertilizer |  |
| M1 | Seedling stage | 30.19 | 25.48 | 33.33 | / | / | / | 2 |
|  | Vigorous growth stage | 30.19 | 64.61 | 66.67 |  |  |  | 4 |
|  | Flower bud differentiation stage | 30.19 | 64.61 | 133.33 |  |  |  | 3 |
|  | Bud development stage | 30.19 | 84.18 | 166.67 |  |  |  | 4 |
|  | Budd emergence stage | 22.64 | 68.02 | 200.00 |  |  |  | 3 |
|  | Fruit expansion stage | 7.55 | 16.15 | 66.67 |  |  |  | 2 |
| M2 | Transplanting stage | 0.00 | 0.00 | 0.00 | / | 3834.65 | / | 1 |
|  | Seedling stage | 12.28 | 22.72 | 21.55 |  | / | 136.61 | 2 |
|  | Vigorous growth stage | 11.98 | 51.13 | 43.10 |  |  | 273.21 | 4 |
|  | Flower bud differentiation stage | 11.98 | 51.13 | 103.32 |  |  | 273.21 | 3 |
|  | Bud development stage | 11.83 | 65.33 | 129.15 |  |  | 341.52 | 4 |
|  | Budd emergence stage | 8.84 | 52.55 | 163.54 |  |  | 273.21 | 3 |
|  | Fruit expansion stage | 3.00 | 12.78 | 55.94 |  |  | 68.3 | 2 |
| M3 | Seedling stage | 34.78 | 7.91 | 33.33 | 18.84 | / | / | 1 |
|  | Vigorous growth stage | 34.78 | 29.43 | 66.67 | 37.67 |  |  | 1 |
|  | Flower bud differentiation stage | 34.78 | 29.43 | 133.33 | 37.67 |  |  | 1 |
|  | Bud development stage | 34.78 | 40.19 | 166.67 | 47.09 |  |  | 1 |
|  | Budd emergence stage | 26.09 | 32.84 | 200.00 | 37.67 |  |  | 1 |
|  | Fruit expansion stage | 8.70 | 7.36 | 66.67 | 9.42 |  |  | 1 |
